# Seroprevalence of Antibodies to Filoviruses with Outbreak Potential in Sub-Saharan Africa: A Systematic Review to Inform Vaccine Development and Deployment

**DOI:** 10.3390/vaccines12121394

**Published:** 2024-12-11

**Authors:** Christopher S. Semancik, Hilary S. Whitworth, Matt A. Price, Heejin Yun, Thomas S. Postler, Marija Zaric, Andrew Kilianski, Christopher L. Cooper, Monica Kuteesa, Sandhya Talasila, Nina Malkevich, Swati B. Gupta, Suzanna C. Francis

**Affiliations:** 1 IAVI, 125 Broad St, New York, NY 10004, USA; 2 Department of Public Health and Community Medicine, Tufts University School of Medicine, Boston, MA 02111, USA; 3 Department of Epidemiology and Biostatistics, University of California at San Francisco, San Francisco, CA 94143, USA; 4 Vaccine Design and Development Laboratory, IAVI, Brooklyn, NY 11220, USA; 5 Department of Infectious Disease Epidemiology, London School of Hygiene and Tropical Medicine, London WC1E 7HT, UK

**Keywords:** filoviruses, seroprevalence, human infection, sub-Saharan Africa, systematic review

## Abstract

**Background/Objectives**: Orthoebolaviruses and orthomarburgviruses are filoviruses that can cause viral hemorrhagic fever and significant morbidity and mortality in humans. The evaluation and deployment of vaccines to prevent and control Ebola and Marburg outbreaks must be informed by an understanding of the transmission and natural history of the causative infections, but little is known about the burden of asymptomatic infection or undiagnosed disease. This systematic review of the published literature examined the seroprevalence of antibodies to orthoebolaviruses and orthomarburgviruses in sub-Saharan Africa. **Methods**: The review protocol was registered on PROSPERO (ID: CRD42023415358) and previously published. Eighty-seven articles describing 85 studies were included, of which seventy-six measured antibodies to orthoebolaviruses and forty-one measured antibodies to orthomarburgviruses. **Results**: The results highlight three central findings that may have implications for vaccine development and deployment. First, substantial antibody seropositivity to Ebola virus (EBOV) and Sudan virus (SUDV) was observed in populations from outbreak-affected areas (≤33% seroprevalence among general populations; ≤41% seroprevalence among healthcare workers and close contacts of disease cases). Second, antibody seropositivity to EBOV, SUDV, and Marburg virus (MARV) was observed among populations from areas without reported outbreaks, with seroprevalence ranging from <1 to 21%. Third, in Central and East Africa, MARV antibody seroprevalence was substantially lower than EBOV or SUDV antibody seroprevalence, even in outbreak-affected areas and in populations at a moderate or high risk of infection (with MARV seroprevalence mostly ranging from 0 to 3%). **Conclusions**: Whilst gaps remain in our understanding of the significance of antibody seropositivity in some settings and contexts, these findings may be important in considering target indications for novel filovirus vaccines, in defining study designs and strategies for demonstrating vaccine efficacy or effectiveness, and in planning and evaluating vaccine deployment strategies to prevent and control outbreaks.

## 1. Introduction

Filoviruses encompass a family of nine genera, of which the orthoebolaviruses and orthomarburgviruses are known to infect humans, with the potential to cause viral hemorrhagic fever (VHF) [1,2]. Diseases caused by orthoebolaviruses (Ebola disease [ED]) and orthomarburgviruses (Marburg disease [MD]) are associated with substantial morbidity and mortality, with case fatality rates (CFRs) ranging from ~25 to 90% [3,4,5]. ED and MD outbreaks result from zoonotic spillover events from animal reservoirs and the subsequent spread from person to person through bodily fluids [1].

Human cases and outbreaks of ED or MD have been reported frequently since the diseases were first described approximately 50 years ago, the majority taking place or originating in sub-Saharan Africa [6,7,8,9]. Some outbreaks have also led to cases arising in countries outside of sub-Saharan Africa, demonstrating the pandemic potential of these diseases [6,9]. The 2014–2016 West Africa ED outbreak (caused by Ebola virus [EBOV]) was the largest recorded to date, killing in 11,310 of 28,616 cases in Guinea, Liberia, and Sierra Leone [7]. The largest recorded MD outbreak occurred in Angola in 2004–2005 (caused by Marburg virus [MARV]), killing 227 of the 252 patients identified [6,8]. At the time of writing, there is an MD outbreak ongoing in Rwanda, a country that had not reported any Marburg cases previously [10]. Of note, some recent ED outbreaks have occurred due to a rare phenomenon of viral reactivation in previously infected individuals, representing an important paradigm for future outbreaks [11].

Countermeasures such as vaccines and therapeutics will be instrumental tools for the prevention and control of future ED and MD outbreaks [12]. Two vaccine regimens have been licensed for protection against EBOV: ERVEBO^®^ (Merck, Rahway, NJ, USA) and the heterologous prime-boost Zabdeno/Mvabea strategy (Johnson & Johnson, New Brunswick, NJ, USA) [13]. To date, there are no vaccines that have been licensed for the prevention of other orthoebolaviruses or orthomarburgviruses. However, there are multiple vaccine candidates in development against MARV and Sudan virus (SUDV), including candidates built on the same platforms as the licensed ERVEBO^®^ vaccine and the candidate ChAd3 and ChAdOx1 Ebola vaccines [14,15,16,17,18,19,20]. The unpredictable nature and the typically small size of filovirus outbreaks means that the demonstration of vaccine efficacy through a gold standard randomized controlled trial is unlikely to be a feasible approach. Instead, vaccine developers need to consider alternative routes to licensure [21]. Two monoclonal antibody-based therapeutics are approved and recommended for the treatment of EBOV in humans (Ebanga™ and Inmazeb^®^ [22,23]), but there are no specific treatments for other filoviruses [24].

The evaluation and deployment of vaccines and therapeutics to prevent and control ED and MD outbreaks must be informed by an understanding of the transmission and natural history of the causative infections. This information is fundamental to defining appropriate and feasible clinical vaccine development programs, defining study endpoints and case definitions, selecting appropriate study populations, identifying individuals and populations that should be targeted and prioritized in vaccine deployment, and considering appropriate vaccination strategies in the event of an outbreak [25]. Studying antibody seroprevalence can provide relevant data on infection and transmission within a population. An earlier systematic review reported estimates of EBOV, SUDV, and Bundibugyo virus (BDBV) antibody seroprevalence ranging from 0 to 46% among people (from countries globally) without a history of known ED [26]. In areas affected by disease outbreaks, seroprevalence ranged from <1 to 17%; in reportedly unaffected areas, estimates ranged from 0 to 24%. Another systematic review reported antibody seroprevalence estimates ranging from <1 to 22% for orthoebolaviruses and 0 to 3% for orthomarburgviruses from population-based studies comprising healthy individuals [27]. Both reviews were conducted nearly a decade ago, and new data have arisen from numerous recent studies.

In this paper, we report on an updated and expanded systematic review of studies on the seroprevalence of antibodies to orthoebolaviruses and orthomarburgviruses in sub-Saharan Africa, stratified by the virus and the level of the risk of infection. We sought to examine all orthoebolaviruses (EBOV, SUDV, BDBV, Taï Forest virus [TAFV], and Reston virus [RESTV]) and orthomarburgviruses (MARV, and Ravn virus [RAVV]) that are known to infect humans, and, thus, have outbreak or epidemic potential, to provide data that can inform priorities for vaccine development and deployment.

## 2. Materials and Methods

This systematic review of the published and peer-reviewed literature was designed to address two key research questions. First, in sub-Saharan Africa, what is the reported seroprevalence of antibodies to orthoebolaviruses and orthomarburgviruses that cause human infection? For this question, we sought to examine seroprevalence over time, according to the geographical region and the level of the risk of infection, and in conjunction with the existing data on ED and MD outbreaks. The second research question concerned the assays used for the detection of antibodies to orthoebolaviruses and orthomarburgviruses and the characteristics of these assays.

Our systematic review was registered on PROSPERO (registration ID: CRD42023415358) and the methods used have been described previously [28]. The methods are briefly summarized below for the purpose of this paper, and the review has been reported in accordance with the Preferred Reporting Items for Systematic Reviews and Meta-Analyses (PRISMA).

### 2.1. Search Strategy

The search strategy was developed based on the primary research question, according to the Population, Intervention, Comparison, Outcome, Study Design (PICOS) framework [29], (Table S1), and using the Peer Review of Electronic Search Strategies (PRESS) guidelines and the Peer Assessment Form [30]. Briefly, PubMed, Embase, and the Web of Science were searched for articles published up until and including 13 March 2024 using Medical Subject Heading (MeSH) and non-MeSH terms for “Ebola”, “Marburg”, “hemorrhagic fever”, “seroprevalence”, and “epidemiology” (Table S2), with no restriction on language. In addition, the reference lists of relevant reviews and included papers were screened for additional articles that were not identified through the database searches.

### 2.2. Eligibility Criteria and Study Selection

All the search results were double screened by two of four reviewers (CSS, CLC, MAP, and TSP) according to pre-defined eligibility criteria (Table S3). To be eligible for inclusion, the articles had to describe primary research that was conducted in humans in sub-Saharan Africa and had to report seroprevalence for one or more orthoebolaviruses (SUDV, EBOV, RESTV, TAFV, or BDBV) or orthomarburgviruses (MARV or RAVV). Studies reporting seroprevalence only among confirmed ED or MD cases were excluded, as we were interested in assessing seropositivity in individuals with no known prior filoviral disease history.

Eligible study designs included cross-sectional studies, cohort studies, and randomized controlled trials. Case studies/series, case-control studies, and reviews were excluded, but studies measuring seroprevalence within the context of an outbreak investigation were included. In the case of disagreements over eligibility, a tertiary screener (SCF) acted as the tiebreaker. The screening was conducted using Rayyan [31].

### 2.3. Data Extraction

Data were double extracted from the eligible articles by two authors (CSS and HY) using a pre-defined and pre-piloted data extraction form and compared to check for consistency. A third author (HSW) conducted a quality review of the extracted data.

The extracted data included the following: the citation and author details; study dates, location, and design; study population (sample size, demographics and characteristics, other participant descriptions); virus(es) under study; sampling methods and assay(s) used; and numerator and denominator data used to calculate seroprevalence estimates. Additional data were extracted to assess for the risk of bias [32].

### 2.4. Assessment of Quality and Strength of Evidence

The risk of bias was assessed for all the studies included in the review using the JBI Prevalence Critical Appraisal Tool, which evaluates the methodological quality of prevalence studies according to the appropriateness of the sample size and the potential for any information bias, selection bias, coverage error, measurement error, or misclassification [33]. Based on this evaluation, the studies were rated as having a “low risk of bias”, “some concerns”, or a “high risk of bias”.

Additionally, the strength of the evidence was evaluated using the Grading of Recommendations, Assessment, Development, and Evaluations (GRADE) certainty ratings [34]. Each article was rated as having a very low, low, moderate, or high certainty level, based on the following domains: the potential for the risk of bias, imprecision, consistency or inconsistency with other similar studies, indirectness, and publication bias.

Further information on the risk of bias and strength of evidence assessments is provided in our previously published protocol [28].

### 2.5. Data Synthesis and Analysis

Summary characteristics of the studies reported by the included articles were tabulated, and seroprevalence estimates were stratified by the virus, African region (according to the African Development Bank’s classification [35]), and study population group (corresponding to likelihood of infection). Study populations of asymptomatic, healthy individuals (where studies specifically stated that all the participants were apparently healthy) and general populations were considered to be at a low risk of infection; healthcare workers (HCW) and people exposed to wildlife (including studies of hunter-gatherers, bushmeat vendors, and miners) were considered to be at a moderate risk; and close contacts of confirmed disease cases and symptomatic individuals or suspected disease cases were considered to be at a high risk. The level of risk was assigned irrespective of date and geographical location and, thus, represents the hypothetical risk within a population group should the virus in question be in circulation. To standardize reporting, 95% confidence intervals (CI) surrounding the prevalence point estimates were calculated by the authors of this review (even if available in the original article) using the Clopper–Pearson (Exact) method, assuming a binomial distribution. The results are presented over time and by geographical region and in the context of known ED and MD outbreaks. Maps providing a visual representation of these data were created using ArcGIS Pro software (ArcGIS Desktop: Release 10; ESRI 2011).

Forest plots were constructed for studies measuring EBOV, SUDV, and MARV antibody seroprevalence among populations considered to be at a low, moderate, or high risk of viral exposure or infection. The results for other viruses were not presented in forest plots due to small numbers of studies. Where forest plots were constructed, the I^2^ statistic was calculated to quantify the degree of heterogeneity among the component studies; the prevalence estimates were pooled using a fixed effects model if the I^2^ statistic was ≤75% [6]. The forest plots were constructed, and meta-analyses performed, using the “meta” package in R statistical software (v4.3.0; R Core Team 2020).

## 3. Results

**Figure 1:**
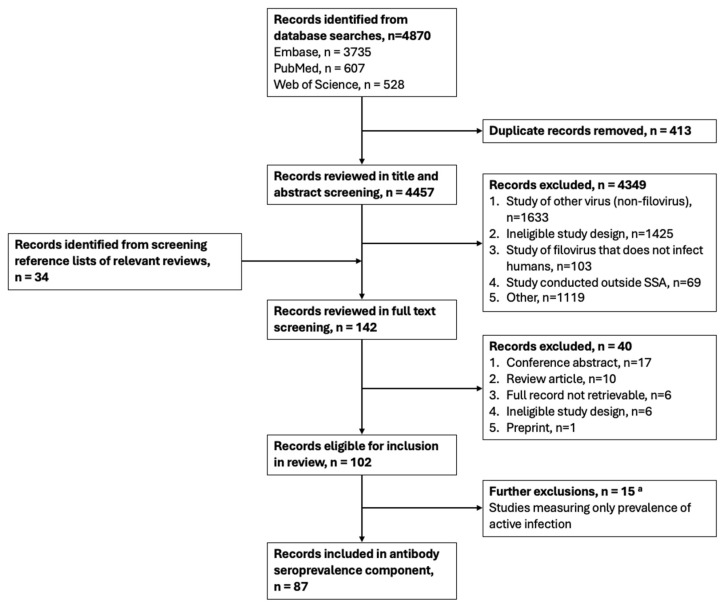
Systematic review flowchart. Abbreviations: n, number; SSA, sub-Saharan Africa. ^a^ Records describing studies that measured the prevalence of active infection (i.e., through viral detection) were eligible for inclusion in the systematic review. However, they were excluded from this paper, which presents the results on antibody seroprevalence (excluded as part of “ineligible study design”). “Other” refers to excluded systematic reviews, methods papers, or non-virus studies that came up during the search.

Overall, 4457 articles were identified through the database searches, of which 87 were eligible for inclusion in our systematic review and described 85 studies that measured antibodies to orthoebolaviruses (n = 76) and/or orthomarburgviruses (n = 41) (Figure 1; Table 1 and Table S4). Articles identified by our review that reported only on the prevalence of active infections (i.e., through viral detection, not antibody seropositivity) are not presented in this paper. Most of the studies were conducted in Central Africa (n = 42; reported in 43 articles), 22 were conducted in West Africa (reported in 23 articles), 14 in East Africa, and 4 in Southern Africa. Three multi-country studies were conducted across African regions. Almost all the identified studies measured IgG responses, either alone or alongside IgM. Only one study measured only IgM responses.

The risk of bias assessments and GRADE ratings for the included studies are shown in Table S5, and details of the assays used for antibody detection (and the criteria to evaluate seropositivity) are provided in Table S6.

### 3.1. Seroprevalence of Antibodies to Ebola Virus (EBOV)

**Figure 2:**
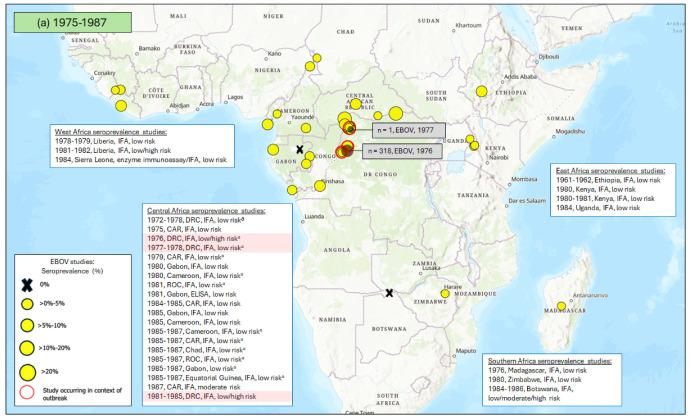

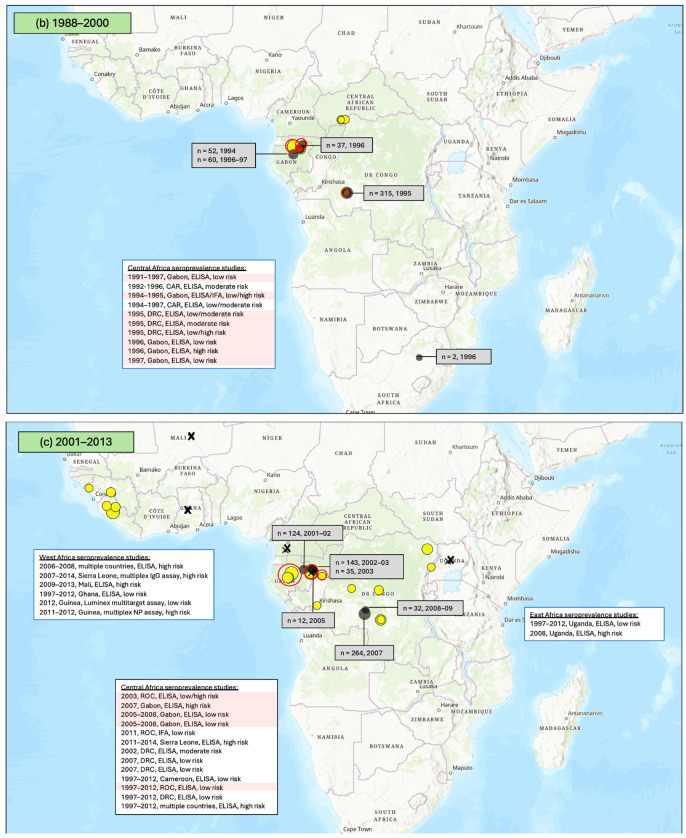

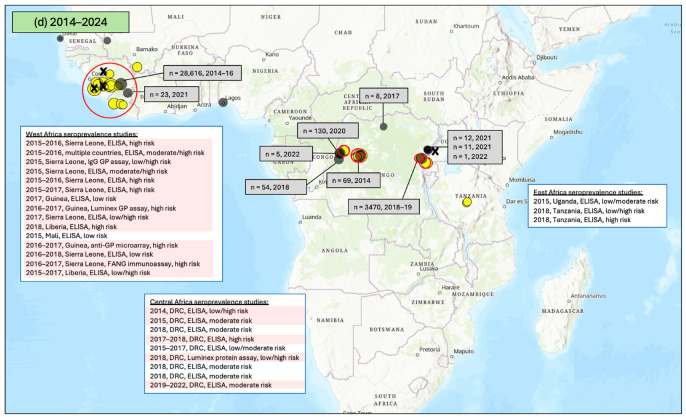
EBOV outbreaks and antibody seroprevalence studies in sub-Saharan Africa from (**a**) 1975 to 1987, (**b**) 1988 to 2000, (**c**) 2001–2013, and (**d**) 2014–2024. The outbreaks are indicated with grey spots and annotated (number of cases, year(s)) with grey boxes. Antibody seroprevalence studies are indicated with yellow spots (or an X if no [i.e., 0%] antibody seropositivity was reported). Brief study details (the dates of sample collection, country/-ies where the study was conducted, type of assay used, and risk level of study population) are provided in white boxes. The red circles and highlighting identify studies that were conducted during or after an EBOV outbreak. Some studies (which are labeled with “^a^”) used assays that measured antibody responses to multiple orthoebolaviruses combined (i.e., they did not distinguish between the antibodies to the different viruses). Where studies were conducted in an area affected by an outbreak of EBOV, we assumed that the seropositivity identified primarily reflects responses to EBOV [36,37,38,39,40,41,42,43,44,45,46,47,48,49,50,51,52,53,54,55,56,57,58,59,60,61,62,63,64,65,66,67,68,69,70,71,72,73,74,75,76,77,78,79,80,81,82,83,84,85,86,87,88,89,90,91,92,93,94,95,96,97,98,99,100,101,102,103,104,105,106,107,108].

The first reported EBOV outbreak in sub-Saharan Africa was in the west of the Democratic Republic of the Congo (DRC, formerly the Republic of Zaire) in 1976 (Figure 2). Since then, the DRC has experienced more outbreaks than any other country, most occurring in the last decade. The only other affected Central African countries to date are Gabon and the Republic of the Congo (ROC).

Despite most EBOV outbreaks occurring in Central Africa, West Africa has seen the most cases. West Africa had never reported any EBOV cases prior to the large multi-country outbreak in 2013–2016, but that outbreak alone was responsible for almost 29,000 cases. No EBOV cases have originated from East Africa. Two cases were reported in South Africa in 1996 (the only cases ever reported in Southern Africa), both of whom had travelled from Central Africa.

Figure 2 and Supplementary Table S4 show the EBOV seroprevalence results from the studies included in this review in regions where EBOV outbreaks were ongoing or had previously been reported and in regions with no documented history of EBOV outbreaks.

#### 3.1.1. East Africa

**Figure 3:**
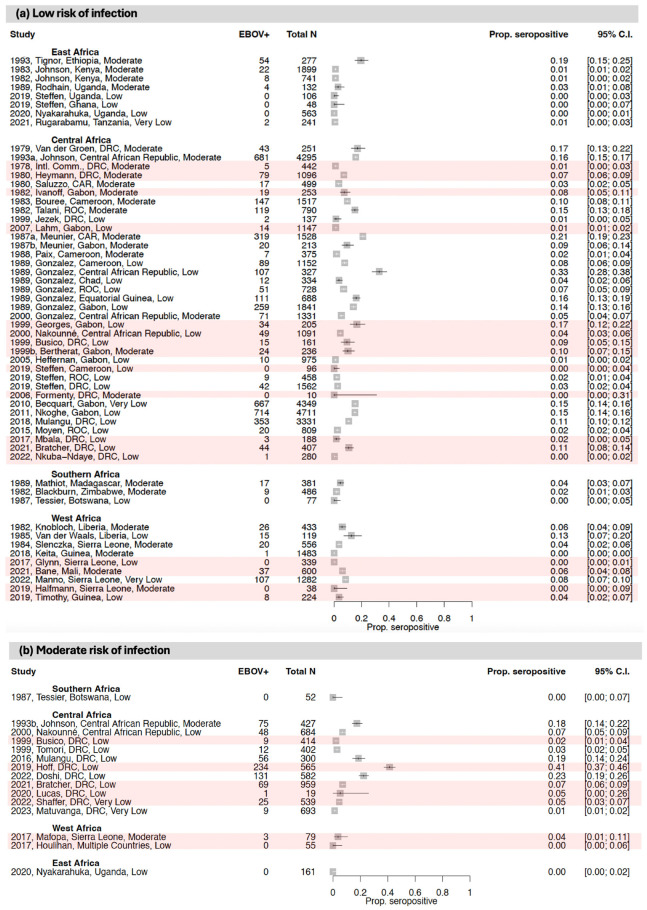

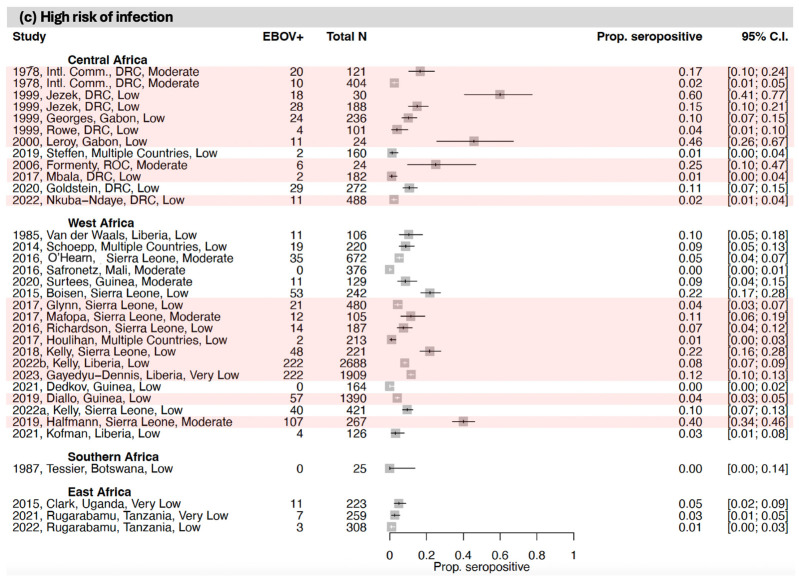
Forest plots showing EBOV antibody seroprevalence with 95% CIs from studies that evaluated population groups at a (**a**) low, (**b**) moderate, or (**c**) high risk of infection. Within each forest plot, the studies are stratified by African region and then ordered by study date (the year that the study was conducted, or the year of sample collection if earlier). The study details provided include the publication date, first author name, study location, and risk of bias rating. For each study, the plot shows the number of individuals who were seropositive (EBOV+) out of the total number sampled. Studies conducted within the context of an EBOV outbreak are shaded in red. Some studies used assays that measured antibody responses to multiple orthoebolaviruses combined (i.e., they did not distinguish between the antibodies to the different viruses) (see Figure 3 and Tables S4 and S6). Where studies were conducted in an area affected by an outbreak of EBOV, we assumed that the seropositivity identified primarily reflects responses to EBOV [36,37,38,39,40,41,42,43,44,45,46,47,48,49,50,51,52,53,54,55,56,57,58,59,60,61,62,63,64,65,66,67,68,69,70,71,72,73,74,75,76,77,78,79,80,81,82,83,84,85,86,87,88,89,90,91,92,93,94,95,96,97,98,99,100,101,102,103,104,105,106,107,108].

Nine studies examined EBOV antibody seroprevalence in East Africa (Figure 2 and Figure 3; Table 1 and Table S4) [36,37,38,39,40,41,42,43,44]. While East Africa has not reported any outbreaks of EBOV to date, two studies were conducted in MD-affected areas, and two were conducted in SUDV-affected areas.

Of the EBOV studies conducted in East Africa, four included an assessment of high-risk-of-infection populations, including symptomatic individuals and close contacts of confirmed or suspected orthoebolavirus cases. In these studies, EBOV seroprevalence ranged from 1.0 to 4.9%, with samples collected between 1980 and 2018. The highest EBOV seroprevalence estimates were reported from a region of Uganda that had previously experienced BDBV outbreaks [43], but there was also apparent seropositivity in Tanzania, which has not reported any orthoebolavirus cases to date [39].

One study included an assessment of moderate-risk-of-infection Ugandan populations (i.e., individuals exposed to wildlife). This study reported 0.0% EBOV seroprevalence in miners in 2015.

Finally, there were five studies that included an assessment of low-risk-of-infection populations, including asymptomatic individuals or general populations. In these studies, EBOV seroprevalence ranged from 0.0 to 19.5%, with samples collected between 1961 and 2018. The highest EBOV seroprevalence estimate was reported from Ethiopia, a country with no documented history of EBOV outbreaks to date [44].

#### 3.1.2. Central Africa

Thirty-nine studies (described in 40 articles) measured EBOV antibody seroprevalence in Central Africa [40,45,46,47,48,49,50,51,52,53,54,55,56,57,58,59,60,61,62,63,64,65,66,67,68,69,70,71,72,73,74,75,76,77,78,79,80,81,82,83]. Twenty were conducted in areas affected by EBOV outbreaks, during or after the outbreaks, of which four included patients with ED symptoms.

Of the EBOV studies conducted in Central Africa, nine included an assessment of high-risk-of-infection populations, including symptomatic individuals and suspected orthoebolavirus cases and close contacts of confirmed or suspected orthoebolavirus cases. In these studies, EBOV seroprevalence ranged from 1.1 to 60.0%, and sample collection occurred between 1976 and 2018. The studies with the highest EBOV seroprevalence estimates came from regions of the DRC and Gabon with prior documented EBOV outbreaks [45,57].

Ten studies included an assessment of moderate-risk-of-infection populations, including healthcare workers and individuals exposed to wildlife. In these groups, EBOV seroprevalence ranged from 2.2 to 41.4%, and sample collection occurred between 1987 and 2020. The highest EBOV seroprevalence estimates were from regions of the DRC that had previously experienced EBOV outbreaks [51,54].

Finally, there were twenty-six studies that included an assessment of low-risk-of-infection populations, including asymptomatic individuals or general populations. In these studies, EBOV seroprevalence ranged from 0.0 to 20.9%, and sample collection occurred between 1972 and 2018. The highest EBOV low-risk seroprevalence estimates came from regions of the Central African Republic and the Republic of the Congo that did not have any documented history of EBOV outbreaks at the time the samples were collected [75,77].

#### 3.1.3. West Africa

Twenty-three studies measured EBOV antibody seroprevalence in West Africa; thirteen in areas affected by the 2013–2016 outbreak, during or shortly after the outbreak [40,84,85,86,87,88,89,90,91,92,93,94,95,96,97,98,99,100,101,102,103,104,105].

Of the EBOV studies conducted in West Africa, seventeen included an assessment of high-risk-of-infection populations, including symptomatic individuals and suspected orthoebolavirus cases and close contacts of confirmed or suspected orthoebolavirus cases. In these studies, EBOV seroprevalence ranged from 0.0 to 40.1%, and samples were collected between 1981 and 2017. The highest EBOV seropositivity estimates came from regions of Sierra Leone with documented EBOV outbreaks [87,89], but multiple studies also showed considerable EBOV seropositivity in Sierra Leone and Guinea before the West African EBOV outbreak began [97,105].

Two studies included an assessment of moderate-risk-of-infection populations, including healthcare workers and individuals exposed to wildlife. In these groups, EBOV seroprevalence ranged from 0.0 to 3.8%, and sample collection occurred between 1987 and 2020.

Finally, there were nine studies that included an assessment of low-risk-of-infection populations, including asymptomatic individuals or general populations. In these studies, EBOV seroprevalence ranged from 0.0 to 12.6%, and sample collection occurred between 1978 and 2018. The highest EBOV seroprevalence estimate came from Liberia in 1981–1982, before the West African EBOV outbreak began [98].

#### 3.1.4. Southern Africa

Three studies were conducted measuring EBOV seroprevalence in Southern Africa [106,107,108]. Of these, one included an assessment of high-risk-of-infection populations (i.e., symptomatic individuals). This study reported 0.0% EBOV seroprevalence in symptomatic individuals in Botswana between 1984 and 1986.

One study included an assessment of moderate-risk-of-infection populations (i.e., people exposed to wildlife). This study reported 0.0% EBOV seroprevalence among individuals exposed to wildlife in Botswana between 1984 and 1986.

Finally, there were three studies that included an assessment of low-risk-of-infection populations, including asymptomatic individuals or general populations. In these studies, EBOV seroprevalence ranged from 0.0 to 4.5%, and sample collection occurred in 1976 and 1980. The highest EBOV seroprevalence estimate came from Madagascar, where there has been no EBOV documented to date [106].

### 3.2. Seroprevalence of Antibodies to Other Orthoebolaviruses

**Figure 4:**
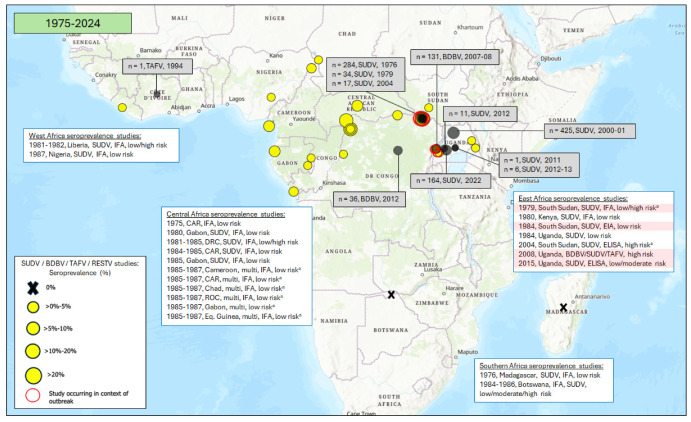
SUDV, BDBV, and TAFV outbreaks and antibody seroprevalence studies in sub-Saharan Africa from 1975 to 2024. Outbreaks are indicated with grey spots and annotated (number of cases, causative virus(es), year(s)) with grey boxes. No human RESTV infections have been reported in sub-Saharan Africa to date. Antibody seroprevalence studies are indicated with yellow spots (or an X if no antibody seropositivity was reported). Brief study details (the dates of sample collection, country/-ies where the study was conducted, type of assay used, and risk level of study population) are provided in white boxes. The red circles and highlighting identify studies that were conducted during or after an outbreak. ^a^ Some studies used assays that measured antibody responses to multiple orthoebolaviruses combined (i.e., they did not distinguish between the antibodies to the different viruses). Where studies were conducted in an area affected by an outbreak of SUDV, we assumed that the seropositivity identified primarily reflects responses to SUDV [37,41,42,43,53,60,64,72,77,82,98,102,103,104,106,108,109,110].

Unlike EBOV, most outbreaks of other orthoebolaviruses in sub-Saharan Africa have been relatively clustered geographically (Figure 4). All reported SUDV outbreaks to date have occurred in East Africa (in South Sudan [close to the DRC border] and Uganda). BDBV outbreaks have been reported on the Uganda/DRC border and in the northeast of the DRC (Central Africa). A single TAFV case was reported in West Africa (specifically, the Côte D’Ivoire) in 1994. No human RESTV cases have been reported in sub-Saharan Africa.

Figure 4 and Supplementary Table S4 show the SUDV, BDBV and TAFV seroprevalence results from studies included in this review in regions where outbreaks of these viruses were ongoing or had previously been reported and in regions with no documented history of these outbreaks.

#### 3.2.1. East Africa

**Figure 5:**
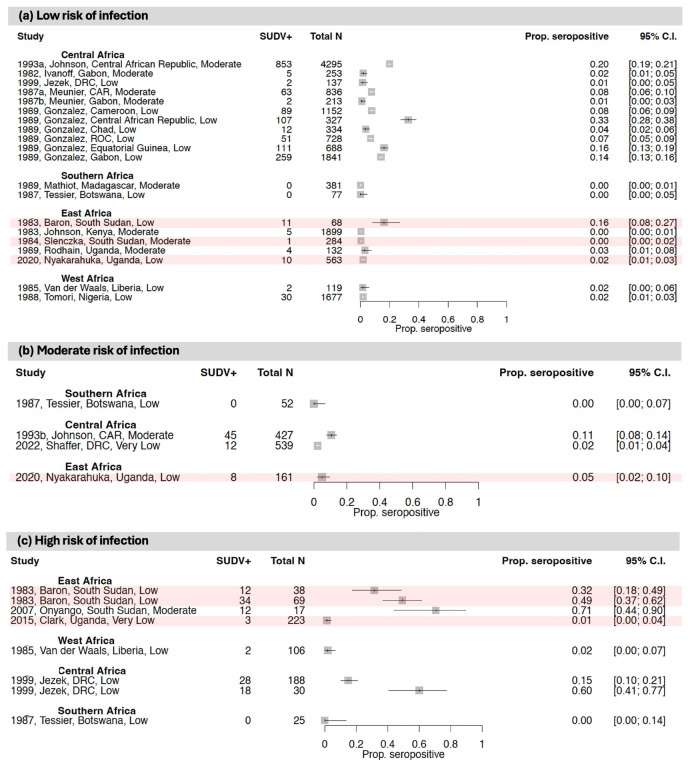
Forest plots showing SUDV antibody seroprevalence with 95% CIs from studies that evaluated population groups at a (**a**) low, (**b**) moderate, or (**c**) high risk of infection. Within each forest plot, the studies are stratified by African region and then ordered by study date (the year that the study was conducted, or the year of sample collection if earlier). The study details provided include the publication date, first author name, study location, and risk of bias rating. For each study, the plot shows the number of individuals who were seropositive (SUDV+) out of the total number sampled. Studies conducted within the context of a SUDV outbreak are shaded in red. Some studies used assays that measured antibody responses to multiple orthoebolaviruses combined (i.e., they did not distinguish between the antibodies to the different viruses) (see Figure 4 and Tables S4 and S6). Where studies were conducted in an area affected by an outbreak of SUDV, we assumed that the seropositivity identified primarily reflects responses to SUDV [37,41,42,43,53,60,64,72,77,82,98,102,103,104,106,108,109,110].

Seven studies examined SUDV and/or BDBV antibody seroprevalence in East Africa (Figure 4 and Figure 5; Table 1 and Table S4) [37,41,42,43,102,109,110]. Three were conducted in South Sudan in areas affected by SUDV outbreaks.

Of the studies conducted in East Africa, three included an assessment of high-risk-of-infection populations, including symptomatic individuals and suspected orthoebolavirus cases and close contacts of confirmed or suspected orthoebolavirus cases. In these studies, SUDV seroprevalence ranged from 1.3 to 70.6%, and samples were collected between 1979 and 2008. The highest SUDV seroprevalence estimates came from regions of South Sudan and Uganda with documented SUDV outbreaks [37,110]. One study in Uganda showed 3.6% BDBV seroprevalence in close contacts of BDBV-confirmed cases in 2008, in the context of a BDBV outbreak [43].

Two studies included an assessment of moderate-risk-of-infection populations, including healthcare workers and individuals exposed to wildlife. In these groups, SUDV seroprevalence ranged from 0.0 to 3.8%, and sample collection occurred between 1979 and 2008. One of the studies also measured 0.0% BDBV seroprevalence in Uganda in individuals exposed to wildlife in 2015.

Finally, there were four studies that included an assessment of low-risk-of-infection populations, including asymptomatic individuals or general populations. In these studies, SUDV seroprevalence ranged from 0.0 to 17.8%, and sample collection occurred between 1979 and 2015. The highest SUDV seropositivity estimate came from South Sudan, where there had been a reported SUDV outbreak, but where no EBOV outbreaks have been documented to date [109]. One study in Uganda showed 0.2% BDBV seroprevalence in a general population in 2015.

#### 3.2.2. Central Africa

Six studies examined SUDV antibody seroprevalence in Central Africa, one of which also measured antibodies to BDBV (Figure 4 and Figure 5; Table 1 and Table S4) [53,60,64,72,77,82]. Of these studies, one included an assessment of high-risk-of-infection populations (i.e., close contacts of suspected cases). This study reported 14.9% SUDV seroprevalence in the DRC between 1981 and 1985.

Two studies included an assessment of moderate-risk-of-infection populations, including healthcare workers and individuals exposed to wildlife. In these groups, SUDV seroprevalence ranged from 2.2 to 15.9%, and sample collection occurred between 1987 and 2018. The highest SUDV seroprevalence estimate came from a region of the Central African Republic which had no documented orthoebolavirus outbreaks at the time of the sample collection [82]. One study reported 2.4% BDBV seroprevalence among healthcare workers in the DRC in 2018.

Finally, there were six studies that included an assessment of low-risk-of-infection populations, including asymptomatic individuals or general populations. In these studies, SUDV seroprevalence ranged from 0.9 to 19.9%, and sample collection occurred between 1980 and 1987. The highest SUDV seroprevalence estimate came from the Central African Republic, where there has been no SUDV documented to date [64].

#### 3.2.3. West Africa

Only three studies were identified from West Africa that measured the seroprevalence of antibodies to orthoebolaviruses other than EBOV (Figure 4 and Figure 5; Table 1 and Table S4) [98,103,104]. Of these, one included an assessment of high-risk-of-infection populations (i.e., symptomatic individuals). This study reported 1.9% SUDV seroprevalence between 1981 and 1982 in Liberia.

Two studies included an assessment of low-risk-of-infection-populations, including asymptomatic individuals. In these studies, SUDV seroprevalence ranged from 1.7 to 1.8%, and sample collection occurred in 1981 and 1987.

#### 3.2.4. Southern Africa

Two studies were conducted to measure SUDV seroprevalence in Southern Africa, where there have been no SUDV outbreaks to date (Figure 4 and Figure 5; Table 1 and Table S4) [106,108]. Of these, one included an assessment of high-risk-of-infection populations (i.e., symptomatic individuals). This study reported 0.0% SUDV seroprevalence between 1984 and 1986 in Botswana in symptomatic individuals.

One study included an assessment of moderate-risk-of-infection populations in Botswana (i.e., individuals exposed to wildlife). This study reported 0.0% SUDV seroprevalence.

Finally, there were two studies that included an assessment of low-risk-of-infection populations, including asymptomatic individuals or general populations. These studies both reported 0.0% SUDV seroprevalence, and sample collection occurred between 1976 and 1986.

### 3.3. Seroprevalence of Antibodies to Orthomarburgviruses

**Figure 6:**
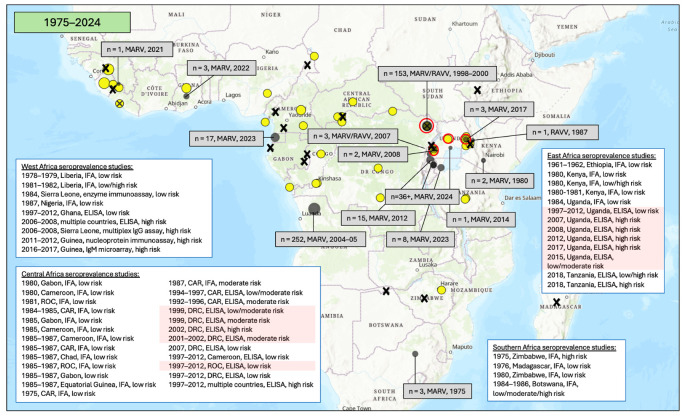
MARV and RAVV outbreaks and antibody seroprevalence studies in sub-Saharan Africa from 1975 to 2024. Outbreaks are indicated with grey spots and annotated (number of cases, causative virus(es), year(s)) with grey boxes. Full details are not yet available for the 2024 Rwandan Marburg outbreak, which is ongoing with 49 confirmed cases and 12 fatalities at the time of writing. The viral sequencing results from the Rwandan outbreak are pending, so the specific causative virus is unknown. Antibody seroprevalence studies are indicated with yellow spots (or an X if no antibody seropositivity was reported). Brief study details (the dates of sample collection, country/-ies where the study was conducted, type of assay used, and risk level of study population) are provided in white boxes. The red circles and highlighting identify studies that were conducted during or after a MARV/RAVV outbreak. The orange circles and shading identify studies that were conducted in areas from where an index case of a MARV outbreak traveled. All studies evaluated MARV antibody seroprevalence [36,37,38,39,41,42,43,44,53,59,60,61,63,64,65,72,75,76,77,81,82,83,84,98,101,102,103,104,105,106,107,108,111,112,113,114,115,116,117,118,119,120,121].

The first MD outbreak recorded in sub-Saharan Africa was in South Africa in 1975 (with the infection suspected to have been imported from Zimbabwe) (Figure 6). Since then, most MD outbreaks have been in East Africa (primarily Uganda) and the east of the DRC. At the time of writing, there is an ongoing outbreak in Rwanda. However, recent years have seen outbreaks also arising in West Africa (in Guinea and Ghana, albeit small), and on the western coast of Central Africa (in Equatorial Guinea). Since 1975, only one additional outbreak has occurred in Southern Africa (in Angola), though this was the largest MD outbreak recorded globally.

Most MD outbreaks have been caused by MARV. A single Kenyan case was caused by RAVV in 1987; both MARV and RAVV were isolated from MD cases during the 2007 Ugandan and 1998–2000 DRC outbreaks. The causative virus of the Rwandan outbreak is not yet confirmed.

Figure 6 and Supplementary Table S4 show the MARV seroprevalence results from the studies included in this review in regions where orthomarburgvirus outbreaks were ongoing or had previously been reported and in regions with no documented history of EBOV outbreaks.

#### 3.3.1. East Africa

**Figure 7:**
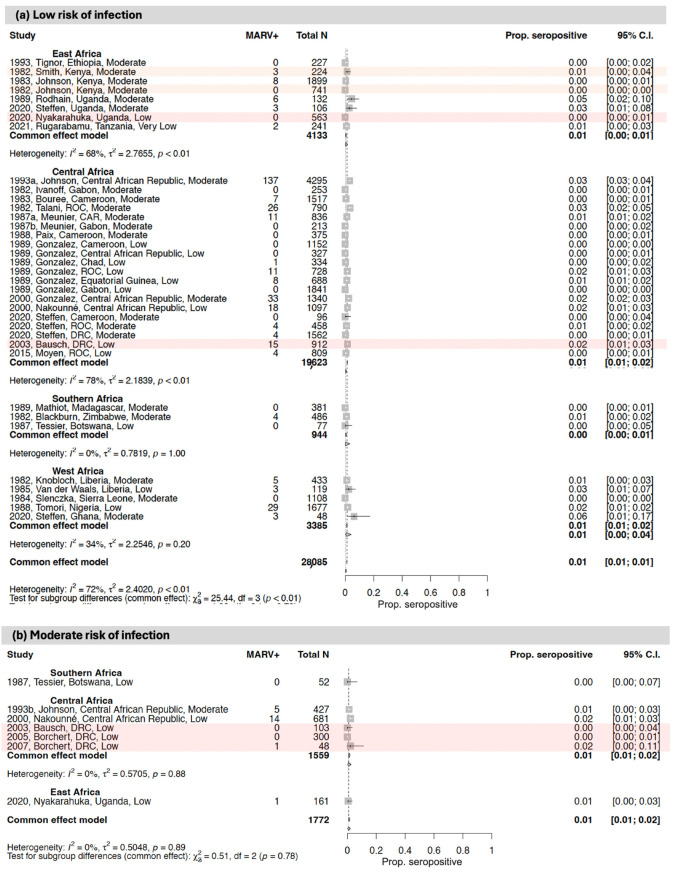

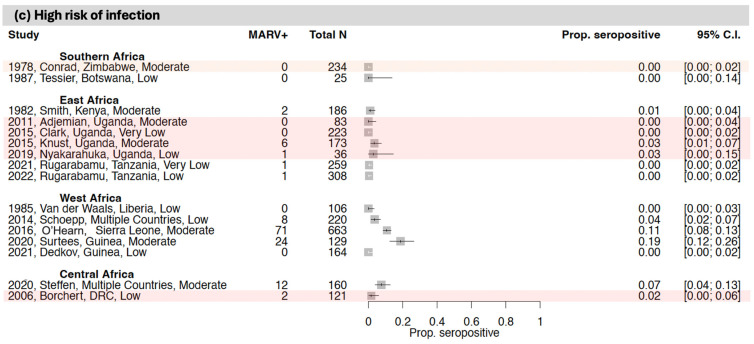
Forest plots showing MARV antibody seroprevalence with 95% CIs from studies that evaluated population groups at a (**a**) low, (**b**) moderate, or (**c**) high risk of infection. Within each forest plot, the studies are stratified by African region and then ordered by the study date (the year that the study was conducted, or the year of sample collection if earlier). The study details provided include the publication date, first author name, study location, and risk of bias rating. For each study, the plot shows the number of individuals that were seropositive (MARV+) out of the total number sampled. Studies conducted within the context of a MARV outbreak are shaded in red. Three studies that were conducted in areas where the index case of a MARV outbreak traveled from are shaded in orange [36,37,38,39,41,42,43,44,53,59,60,61,63,64,65,72,75,76,77,81,82,83,84,98,101,102,103,104,105,106,107,108,111,112,113,114,115,116,117,118,119,120,121].

Fourteen studies evaluated MARV antibody seroprevalence in East Africa, including five in areas of Uganda affected by the 2007 and 2017 MARV outbreaks and three in the region where the index case of the 1980 Kenyan outbreak lived and worked (Figure 6 and Figure 7; Table 1 and Table S4) [36,37,38,39,41,42,43,44,102,111,112,113,114,115].

Of the MARV studies conducted in East Africa, seven included an assessment of high-risk-of-infection populations, including symptomatic individuals and suspected orthomarburgvirus cases and close contacts of confirmed or suspected orthomarburgvirus cases. In these studies, MARV seroprevalence ranged from 0.0 to 3.5%, and samples were collected between 1980 and 2018. The highest MARV seroprevalence estimate came from a study in a region of Uganda that had experienced a previous MARV outbreak [113].

One study included an assessment of moderate-risk-of-infection populations (i.e., individuals exposed to wildlife). This study reported 0.6% MARV seroprevalence in Uganda in 2015.

Finally, there were six studies that included an assessment of low-risk-of-infection populations, including asymptomatic individuals or general populations. In these studies, MARV seroprevalence ranged from 0.0 to 4.5%, and sample collection occurred between 1961 and 2018. The highest MARV seroprevalence estimate came from a study in a region of Uganda that had not experienced any documented MARV outbreaks at the time of the sample collection [41].

#### 3.3.2. Central Africa

Almost half of the studies of MARV antibody seroprevalence to date are from Central Africa, making up a total of nineteen studies (Figure 6 and Figure 7; Table 1 and Table S4) [53,59,60,61,63,64,65,72,75,76,77,81,82,83,115,116,117,118,119]. Of these, one included an assessment of high-risk-of-infection populations (i.e., close contacts of confirmed cases). This study reported 1.7% MARV seroprevalence in household contacts in the DRC in 2002.

Six studies included an assessment of moderate-risk-of-infection populations, including individuals exposed to wildlife and healthcare workers. In this group, MARV seroprevalence ranged from 0.0 to 2.5%, and sample collection occurred between 1987 and 2002. The highest MARV moderate-risk seroprevalence estimate came from the Central African Republic, where there has been no EBOV documented to date [83].

Finally, there were twelve studies that included an assessment of low-risk-of-infection populations, including asymptomatic individuals or general populations. In these studies, MARV seroprevalence ranged from 0.0 to 3.3%, and sample collection occurred between 1980 and 2011. The highest MARV low-risk seroprevalence estimate came from a region of the Republic of the Congo which had not reported any MARV outbreaks to date [75].

#### 3.3.3. West Africa

Eight studies examined MARV antibody seroprevalence in West Africa (Figure 6 and Figure 7; Table 1 and Table S4) [84,98,101,103,104,105,115,120]. Of these, five included an assessment of high-risk-of-infection populations, including symptomatic individuals and suspected orthomarburgvirus cases and close contacts of confirmed or suspected orthomarburgvirus cases. In these studies, MARV seroprevalence ranged from 0.0 to 18.6%, and samples were collected between 1981 and 2017. The highest MARV seroprevalence estimates came from Sierra Leone and Guinea, where there have been no reported MARV outbreaks to date [105,120].

There were three studies that included an assessment of low-risk-of-infection populations, including asymptomatic individuals or general populations. In these studies, MARV seroprevalence ranged from 1.2 to 2.5%, and sample collection occurred between 1978 and 1987. The highest MARV seroprevalence estimate came from Liberia, where there has been no documented MARV to date [98].

#### 3.3.4. Southern Africa

Four studies were conducted to measure MARV seroprevalence in Southern Africa (Figure 6 and Figure 7; Table 1 and Table S4) [106,107,108,121]. Of these, two included an assessment of high-risk-of-infection populations, including symptomatic individuals and suspected orthomarburgvirus cases and close contacts of confirmed or suspected orthomarburgvirus cases. The reported MARV seroprevalence was 0.0% in both surveys, and samples were collected between 1975 and 1986.

One study included an assessment of moderate-risk-of-infection populations in Botswana (i.e., individuals exposed to wildlife). This study reported 0.0% MARV seroprevalence between 1984 and 1986.

Finally, there were three studies that included an assessment of low-risk-of-infection populations, including asymptomatic individuals or general populations. In these studies, MARV seroprevalence ranged from 0.0 to 0.8%, and sample collection occurred between 1976 and 1986. The highest MARV seroprevalence estimate came from a region of Zimbabwe with no documented MARV outbreaks to date [107].

### 3.4. Assays Used for Detection of Antibodies to Orthoebolaviruses and Orthomarburgviruses

Several types of analyte binding methods and virological techniques have been applied to the serology assessments of orthoebolaviruses and orthomarburgviruses described in this review—the most common being immunofluorescence assays (IFAs) (n = 32) and enzyme-linked immunosorbent assays (ELISAs) (n = 50). The breadth of the reported serological assays was large and diverse. We described the features of all the identified assays and indicated performance metrics when provided by the authors (Table S6). It is known that seroprevalence estimates can vary considerably, based on the assay used. Differences in the detection mechanism of serological assays targeting the same virus have been observed in this systematic review, which could have limited the comparability of the seroprevalence estimates for populations being tested. There has also been a noticeable lack of standardization between the assay methodologies.

We found that only a small proportion of independent author groups conducted their own assay pre-study validation before rolling out their serosurvey. Assay performance has direct consequences on the validity of a study, where the assay sensitivity and specificity reflect whether a given seroprevalence result is accurately reflective of the sample’s true antibody positivity. For instance, low specificity assays can lead to overinflated seroprevalence estimates, creating misleading results, particularly in settings with low true prevalences. For filoviral assays, non-specific reactivity to antibodies against related filoviruses can be a particular problem. This problem may be complicated further by the possibility that assays could be cross-reacting with filoviruses that are unknown as of now and not documented in the literature [122,123]. In addition, sensitivity and specificity can vary for the same assay, depending on the samples they were tested with. In some of the included studies, we observed that assay performance characteristics were incompletely evaluated or sometimes not described at all. Only a few author groups described the evaluation panels and methods they used.

Overall, we found that a large and diverse number of assays were used in the seroprevalence studies described in this systematic review; this diverse selection of serological assays, alongside a lack of assessments on assay performance characteristics, may have impacted the interpretation and the reliability of the seroprevalence studies.

## 4. Discussion

This systematic review highlights three central findings that may have implications for vaccine development and deployment. First, there is a demonstrated history of considerable antibody seropositivity to EBOV and SUDV in populations from areas affected by outbreaks of these viruses. This was observed in groups considered to be at a moderate or high risk of infection, such as HCWs and close contacts of confirmed ED cases (up to 46% EBOV seroprevalence in Central and West Africa; up to 25% EBOV/SUDV seroprevalence in East Africa), but also in groups considered to be at a lower risk, namely general population groups (up to 21% EBOV seroprevalence in Central and West Africa; up to 20% EBOV/SUDV seroprevalence in East Africa). While seropositivity is not conclusive evidence of infection, this finding raises the possibility that a substantial number of asymptomatic infections or mild/moderate disease cases remain undiagnosed during ED outbreaks, which may have the potential to contribute to viral transmission. An earlier systematic review estimated that 27% of Ebola infections are asymptomatic [124], but our results suggest that this proportion could potentially be somewhat higher.

Second, seropositivity to EBOV, SUDV, and, to a lesser extent, MARV has been consistently reported among populations from areas seemingly unaffected by these viruses (either to date, or at the time the studies were conducted). In some studies, seroprevalence was very low (for example, 1–3% for EBOV in most studies from East Africa, where no confirmed EBOV cases had been reported to date). This could represent infections that occurred elsewhere, prior to the participant migrating into the study area. However, perhaps more likely, this may reflect false positive test results. Many other studies detected substantial antibody seropositivity, particularly for EBOV in Central and West Africa, for SUDV in Central Africa, and for MARV in West Africa. In some cases, antibody seroprevalence was remarkably high (for example, 32% for EBOV/SUDV in the CAR [65]). This substantial seropositivity raises a possibility of viral exposure, asymptomatic infection, or undiagnosed disease in the study areas, potentially implying that the geographic footprint of the viruses (and, thus, the area at risk of future outbreaks) is more widespread than indicated by the case reporting to date.

Third, in Central and East Africa (the two African regions with the most MARV outbreaks to date), MARV antibody seroprevalence appears to be much lower than EBOV or SUDV seroprevalence. This was observed in populations from areas affected by outbreaks, regardless of the risk of viral exposure or infection (≤3% MARV antibody seroprevalence among close contacts of MD cases, HCWs, and general populations), as well as in reportedly unaffected areas (also ≤3% seroprevalence in most studies). Whilst this could indicate a lower risk of viral exposure or transmission for MARV compared to EBOV and SUDV, this is not supported by earlier studies [124,125]. Another explanation may be a lower false-positive rate for the MARV antibody tests than for the EBOV/SUDV antibody tests. Notably, however, substantial MARV antibody seropositivity was reported in West Africa (4–19% seroprevalence in most studies), despite the region reporting very few MD cases to date.

The possibility of a significant burden of asymptomatic or mild filoviral infection, which could have the potential to contribute to viral transmission, may have implications for defining the target indication and the profile of novel filoviral vaccines, with a potent ability to reduce viral transmission perhaps being prioritized alongside the prevention of severe disease and death. This, in turn, has implications for vaccine efficacy/effectiveness study design and endpoints and for the modeling of the projected impact of vaccination. The identification of populations with high filoviral seropositivity (if truly reflective of viral exposure and infection) may also affect considerations surrounding appropriate populations in which to conduct efficacy or effectiveness evaluations. On the one hand, the data may help vaccine developers to strategize and identify countries and areas that may be at a risk of future outbreaks (alongside other data, for example on the geographical distribution of natural reservoir animal species), thus facilitating preparedness for conducting reactive studies should cases arise. On the other hand, high baseline seropositivity could have implications for efficacy/effectiveness and immunogenicity assessments that may need to be considered. Finally, the findings of this review may be informative for vaccine developers, policymakers, and other stakeholders in consideration of appropriate, impactful, and cost-effective vaccine deployment strategies, including the identification of population groups that should be prioritized and targeted for vaccination and the consideration of methods of deployment such as ring vaccination.

Our review also identified several important evidence gaps. First, there are few data from Southern Africa for any of the viruses, despite a history of EBOV and MARV cases being reported in this region. Notably, the largest reported MARV outbreak to date was in Angola. Second, there are few studies that have examined the seroprevalence of antibodies to orthoebolaviruses and orthomarburgviruses other than EBOV, SUDV, and MARV. While BDBV, TAFV, and RAVV outbreaks have been rare to date, it is important to understand and monitor their potential for future outbreaks, and seroprevalence data may be informative for vaccine developers considering a pan-filovirus vaccine. RESTV (most closely related to SUDV) has not been shown to cause disease in humans, but asymptomatic infection in a human exposed to infected pigs in the Philippines has been reported. Thus, monitoring for emergent and potentially human-pathogenic variants may be warranted [126].

The findings of this review should be considered within the context of the observed limitations of the included studies. In general, there were few large, well-designed, and well-described seroprevalence studies, as reflected by the small number of studies assigned a low or very low risk of bias rating. Most studies did not use probability sampling methods or did not adequately describe how the participants were sampled. In some cases, this was appropriate (for example, studies that purposively sampled all close contacts of confirmed ED or MD cases within the context of an outbreak investigation). However, for most studies, the lack of random sampling likely resulted in a study population that is not representative of the target population and, thus, estimates that are impacted by selection biases. Many articles did not adequately describe the study population, so the potential impact of biases was often difficult to evaluate. Some studies were limited by very small sample sizes, resulting in wide confidence intervals surrounding seroprevalence point estimates.

All the studies conducted before 1990 used IFA for antibody detection, an assay which is now notorious for low sensitivity and specificity, potentially introducing measurement errors and misclassifications. Even with later, more advanced assays, false positive results are frequently reported, and non-specific reactivity to antibodies against related filoviruses can be a particular problem [122,123,127,128,129]. Furthermore, the approaches to assay cutoff determination and definitions for a seropositive response varied considerably across studies. This means that the definition of what was considered a “seropositive sample” varied widely from study to study, even in those studies that used similar assays. Notably, it is possible that other endemic infections, such as malaria, may have caused elevated levels of various inflammatory markers that could have negatively impacted assay sensitivity and specificity [130]. These limitations frequently lead to uncertainty in the meaning and significance of observed antibody seropositivity. Furthermore, while an evaluation of the quality of sample collection, storage, and processing, as well as laboratory conditions and practices, was outside the scope of this review, these factors can have substantial impacts on the reliability of results. Well-designed seroprevalence studies are hugely valuable for evaluating previous infections and current levels of immunity in target populations. However, an understanding of the uncertainties and limitations of existing studies is crucial to the appropriate interpretation of results.

The methodology and results of our systematic review also have several limitations. First, due to the high heterogeneity between the included studies, it was often not possible to conduct meta-analyses and provide pooled seroprevalence estimates. Second, whilst participant age is likely to be an important factor influencing antibody seropositivity, and it would be valuable to better understand infection and transmission by age group, we were not able to systematically assess or stratify the results by age because of considerable variation in how the studies reported and described the age of their study populations and seroprevalence across age groups.

Despite these limitations, there are notable strengths and benefits of our review. The filovirus landscape has evolved in sub-Saharan Africa in recent years (since the earlier reviews were conducted [26,27]), with the number of EBOV outbreaks doubling in the last decade and with the spread of both EBOV and MARV to West Africa. Our review provides a comprehensive update to the existing evidence base on filoviral seroprevalence in sub-Saharan Africa and builds on the earlier reviews through the evaluation of all filoviruses with outbreak potential in humans. Furthermore, by classifying the study populations according to the risk of infection and interpreting the results by geographical region and in the context of ED and MD outbreaks, our review provides a better understanding of where, when, and among whom seropositive results are reported. Through this stratification, we also observed patterns of internal consistency among the studies, despite inconsistencies between the component studies. For example, on the whole, we saw higher filovirus seropositivity rates in high-risk groups than in low-risk groups.

Our review highlights a clear and urgent need for more well-designed studies examining filovirus seroprevalence across all the regions of sub-Saharan Africa, using high-quality and standardized methods for antibody detection and measurement. We also need to better understand any role of potential asymptomatic filovirus infections and undiagnosed diseases in viral transmission, as well as the potential sources and routes of viral exposure and transmission in populations with substantial antibody seropositivity. A review of the evidence on filovirus infections in animals (wild and domestic) across sub-Saharan Africa and the potential for spillover events would be valuable. Finally, a key question arising from this systematic review is why there appears to be extensive filoviral seroprevalence in areas without a history of reported ED and MD cases and the meaning of this apparent seropositivity.

## Figures and Tables

**Table 1:** Summary of antibody seroprevalence studies, identified by virus and population group. Data indicate numbers of studies, unless specified otherwise.

Population Group	Variable	Studies Conducted					
		EBOV	SUDV	BDBV	RESTV	TAFV	MARV
Asymptomatic, healthy individuals (low risk)	Total N ^a^	11	6	0	0	0	5
	African region ^b^			-	-	-	
	East	1	2				1
	Central	6	1				1
	West	3	2				2
	Southern	1	1				1
	Outbreak area ^c^			-	-	-	
	Yes	6	2				0
	No	5	4				5
	Seroprevalence (%) ^d^	0.0–15.3	0.0–17.8	-	-	-	0.0–4.5
General population (low risk)	Total N ^a^	33	8	1	0	0	20
	African region ^b^				-	-	
	East	3	2	1			7
	Central	21	5	0			10
	West	6	0	0			1
	Southern	2	1	0			2
	Multi-region	1	0	0			0
	Outbreak area ^c^				-	-	
	Yes	15	2	0			3
	No	18	6	1			17
	Seroprevalence (%) ^d^	0.0–20.9	0.0–19.9	0.2	-	-	0.0–3.2
Healthcare workers ^e^ (moderate risk)	Total N ^a^	8	1	1	0	0	2
	African region ^b^				-	-	
	East	0	0	0			0
	Central	6	1	1			2
	West	2	0	0			0
	Outbreak area ^c^				-	-	
	Yes	8	1	1			2
	No	0	0	0			0
	Seroprevalence (%) ^d^	0.0–41.4	2.2	2.4	-	-	0.0–2.1
People exposed to wildlife ^f^ (moderate risk)	Total N ^a^	7	4	2	0	1	6
	African region ^b^				-		
	East	1	1				1
	Central	5	2	1		0	4
	West	0	0	1		1	0
	Southern	1	1	0		0	1
	Outbreak area ^c^				-		
	Yes	3	2	2		1	3
	No	4	2	0		0	3
	Seroprevalence (%) ^d^	0.0–18.7	0.0–10.5	0.0–5.2	-	5.2	0.0–5.2
Close contacts of confirmed cases (high risk)	Total N ^a^	18	3	1	0	1	8
	African region ^b^				-		
	East	2	2	1		1	6
	Central	6	1	0		0	1
	West	10	0	0		0	0
	Southern	0	0	0		0	1
	Outbreak area ^c^				-		
	Yes	17	3	1		0	5
	No	1	0	0		1	3
	Seroprevalence (%) ^d^	0.9–45.8	1.3–32.0	3.6	-	0.4	0.0–3.5
Symptomatic/Suspected Cases ^g^ (high risk)	Total N ^a^	20	6	1	1	0	10
	African region ^b^					-	
	East	3	1	0	0		2
	Central	5	2	1	1		1
	West	10	1	0	0		5
	Southern	1	1				1
	Multi-region	1	0	0	0		1
	Outbreak area ^c^					-	
	Yes	7	2	0	0		0
	No	13	4	1	1		10
	Seroprevalence (%) ^d^	0.0–70.6	0.0–70.6	0.4	0.0	-	0.0–18.6

Abbreviations: EBOV, Ebola virus; SUDV, Sudan virus; BDBV, Bundibugyo virus; RESTV, Reston virus; TAFV, Taï Forest virus; MARV, Marburg virus; N, number. ^a^ Total number of studies conducted for each virus and population group. Some studies looked at multiple viruses and/or multiple population groups, so the sum of the total numbers of studies across the categories is >87. ^b^ Numbers of studies conducted in East, Central, or West Africa. ^c^ The number of studies conducted in areas affected by an outbreak of the virus being evaluated (either at the time of the study or previously) versus studies conducted in areas where an outbreak had not previously been reported. ^d^ Reported antibody seroprevalence. The range of seroprevalence point estimates (min–max) is provided where >1 contributing study was identified. ^e^ In this study, “healthcare workers” included clinical and non-clinical workers at healthcare facilities, informal care givers (such as traditional healers, pastors, etc.), and other staff involved in a healthcare setting or response. ^f^ In this study, “people exposed to wildlife” included hunter–gatherers (n = 5), bushmeat vendors (n = 1), miners (n = 1), and individuals that otherwise have exposure to wildlife (n = 2). ^g^ Not clinically confirmed.

## Data Availability

Data may be made available upon reasonable request to the corresponding author and upon agreement by the co-authors, IAVI, and the funder.

## Supplementary Materials

The following supporting information can be downloaded at: https://www.mdpi.com/article/10.3390/vaccines12121394/s1. Table S1: development of search strategy using PICOS framework; Table S2: search strategy for each database; Table S3: inclusion and exclusion criteria; Table S4: studies measuring seroprevalence of antibodies to orthoebolaviruses or orthomarburgviruses; Table S5: risk of bias assessment and GRADE rating for studies measuring antibody seroprevalence; and Table S6: assays used for detection of antibodies against orthoebolaviruses and orthomarburgviruses in antibody seroprevalence studies.
