# Supplementary material for: Seroprevalence of Antibodies to Filoviruses with Outbreak Potential in Sub-Saharan Africa: A Systematic Review to Inform Vaccine Development and Deployment
Source: Vaccines (Basel). 2024 Dec 11;12(12):1394. doi: 10.3390/vaccines12121394 (PMC11726543; doi:10.3390/vaccines12121394)
Supplement: Supplementary file 1 [file vaccines-12-01394-s001.zip › vaccines-3279176-supplementary.pdf]

Supplementary Material

Seroprevalence of Antibodies to Filoviruses with Outbreak Potential in Sub-Saharan Africa: A Systematic Review to Inform Vaccine Development and Deployment

Christopher S. Semancik [1,2,†], Hilary S. Whitworth [1,*,†], Matt A. Price [1,3], Heejin Yun [1], Thomas S. Postler [4],
Marija Zaric [1], Andrew Kilianski [1], Christopher L. Cooper [4], Monica Kuteesa [1], Sandhya Talasila [1],
Nina Malkevich [1], Swati B. Gupta [1] and Suzanna C. Francis [1,5]

[1] IAVI, 125 Broad St, New York, NY 10004, USA; christopher.semancik@tufts.edu (C.S.S.); mprice@iavi.org (M.A.P.); hyun@iavi.org (H.Y.); mzaric@iavi.org (M.Z.); akilianski@gmail.com (A.K.);
mkuteesa@iavi.org (M.K.); stalasila@iavi.org (S.T.); nmalkevich@iavi.org (N.M.); sgupta@iavi.org (S.B.G.); sfrancis@iavi.org (S.C.F.)
[2] Department of Public Health and Community Medicine, Tufts University School of Medicine,
Boston, MA 02111, USA
[3] Department of Epidemiology and Biostatistics, University of California at San Francisco, San Francisco, CA 94143, USA
[4] Vaccine Design and Development Laboratory, IAVI, Brooklyn, NY 11220, USA; tpostler@iavi.org (T.S.P.); ccooper@iavi.org (C.L.C.)
[5] Department of Infectious Disease Epidemiology, London School of Hygiene and Tropical Medicine,
London WC1E 7HT, UK
* Correspondence: hwhitworth@iavi.org
† These authors contributed equally to this work.

SUPPLEMENTARY MATERIAL

Table S1. Development of search strategy using PICOS framework.

**P**opulation	People living in sub-Saharan Africa
**I**ntervention	No intervention
**C**omparison	No comparison; prevalence stratified by population characteristics will be extracted
**O**utcome [a]	Confirmed presence of virus or antibodies for EBOV, SUDV, BDBV, TAFV, RESTV, MARV, or RAVV, as measured by either viral testing (e.g., RT-PCR) or antibody testing (e.g., ELISA)

**S**tudy Design	Cross-sectional studies, cohort studies, and randomized controlled trials; no case studies or case-control studies

[a] Studies that examined prevalence of active infection were identified through our systematic review but excluded in this paper, which is focused on antibody seroprevalence.

Table S2. Search strategy for each database.

**Database**	**Search Terms**
PubMed (PubMed interface)	1. "Ebolavirus" [MeSH]<br>2. "Hemorrhagic Fever, Ebola" [MeSH]<br>3. "Marburgvirus" [MeSH]<br>4. Hemorrhagic fever*[tiab]<br>5. Ebola[tiab]<br>6. Ebolavirus*[tiab]<br>7. Marburg virus*[tiab]<br>8. Frankfurt Marburg syndrome virus[tiab]<br>9. (1 OR 2 OR 3 OR 4 OR 5 OR 6 OR 7 OR 8)
	10. "Seroepidemiologic Studies" [MeSH:NoExp]<br>11. "Epidemiologic Methods" [MeSH:NoExp]<br>12. Seroprevalence*[tiab]<br>13. Serology[tiab]<br>14. Seroepidemiolog*[tiab]<br>15. (10 OR 11 OR 12 OR 13 OR 14)
	16. ((9) AND (15))
Embase (Elsevier interface)	1. 'Ebola hemorrhagic fever'/exp<br>2. 'Marburgvirus'/exp<br>3. 'Ebolavirus'/exp<br>4. 'Marburg hemorrhagic fever'/exp<br>5. Ebola*<br>6. Marburg*<br>7. (1 OR 2 OR 3 OR 4 OR 5 OR 6)
	8. 'Seroepidemiology'/exp<br>9. 'Seroprevalence'/exp<br>10. 'Epidemiological surveillance'/exp<br>11. 'Prevalence'/exp<br>12. Seroepidemiol*<br>13. Seroprevalence*<br>14. Surveil*<br>15. (8 OR 9 OR 10 OR 11 OR 12 OR 13 OR 14)
	16. [humans]/ilm

	17. ((7) AND (15) AND (16))
Web of Science (Clarivate interface)	1. Ebolavirus<br>2. Marburgviruses<br>3. Ebola*<br>4. Ebolavirus*<br>5. Marburg*<br>6. Marburgviruses*<br>7. (1 OR 2 OR 3 OR 4 OR 5 OR 6)
	8. Seroepidemiol*<br>9. *Prevalence*<br>10. Seroprevalence*<br>11. Serology<br>12. Seroepidemiolog*<br>13. (8 OR 9 OR 10 OR 11 OR 12)
	14. (TS = (7)) AND TS = (13))

Table S3. Inclusion and exclusion criteria.

Inclusion Criteria	Exclusion Criteria
Human research studies	Studies with non-human animals
Cohort studies, cross-sectional studies, randomized controlled trials	Case-control studies
Studies conducted in sub-Saharan Africa of populations without a confirmed ED or MD diagnosis	Studies conducted outside of sub-Saharan Africa, or only in populations with confirmed ED or MD diagnoses
Studies on orthoebolaviruses (EBOV, SUDV, TAFV, BDBV, RESTV) or orthomarburgviruses (MARV, RAVV) that have been shown to infect humans	Studies on filoviruses that have not been shown to cause infection in humans (i.e., BOMV and filoviruses other than orthoebolaviruses and orthomarburgviruses)
All assays for diagnosis, including RT-PCR, ELISA, or any other laboratory assay measuring antibody or filovirus antigen[a]	
Individuals of all ages	
Individuals of all occupations and exposure statuses	

[a] Studies that examined prevalence of active infection were identified through our systematic review but excluded in this paper, which is focused on seroprevalence.

Table S4. Studies measuring seroprevalence of antibodies to orthoebolaviruses or orthomarburgviruses. Studies are grouped by African region and ordered by date of publication. Those conducted during or after an outbreak of (one of) the virus(es) evaluated are shaded in red; those conducted in an area where an outbreak index case travelled from are shaded in orange.

Publication date (study date[a]) Author, Location	Study population – Population category[b]: Details	Study design, Sampling method[c]	Sample size (% M)	Participant age – Range, ave, IQR/SD	Virus evaluated[d]	Antibody detection assay[e]	Seroprevalence – N seropositive / total N, % (95% CI)[f]
**EAST AFRICA**							
1982 (1980) Smith, Kenya	Close contacts: Close contacts of 2 confirmed MD patients in western Kenya	Cross-sectional, Purposive sampling	410 (% M NS) Close contacts: 186 Gen. popn.: 224	NS	MARV	IFA	2 / 186, 1.1 (0.1-3.8)
	General population: Residents of Nzoia, where the index MD case resided						3 / 224, 1.3 (0.3-3.9)
1982 (1980-81) Johnson, Kenya	Close contacts: Suspected cases and their contacts, hospital staff, and other local populations	VHF surveillance, Purposive sampling	741 (50.6% M)	NS	EBOV MARV	IFA	EBOV: 8 / 741, 1.1 (0.5-2.1)<br>MARV: 0 / 741, 0.0 (0.0-0.5)
1983 (1979) Baron, South Sudan	Close contacts: Asymptomatic relatives of ED cases	Outbreak investigation, Purposive sampling	175 (% M NS) Close contacts: 61 Gen. popn.: 45	NS (all ≥16y)	'Ebola'[g] (assumed to be SUDV, since study in South Sudan)	Indirect IFA	15 / 61, 24.6 (14.5-37.3)<br>Physical contact: 12 / 38, 31.6 (17.5-48.7)<br>No physical contact, 3 / 23, 13.0 (2.8-33.6)
	Asymptomatic, healthy: Asymptomatic relatives of non-ED febrile cases						8 / 45, 17.8 (8.0-32.1)
1983 (1980) Johnson, Kenya	General population: Residents of various regions in Kenya	Cross-sectional, Cluster sampling	1899 (% M NS)	1-70y	EBOV SUDV MARV	IFA	EBOV: 22 / 1899, 1.2 (0.7-1.8)<br>SUDV: 5/ 1899, 0.3 (0.1-0.6)<br>MARV: 8/1899, 0.4 (0.2-0.8)
1989 (1984) Rodhain, Uganda	Asymptomatic, healthy: Apparently healthy adult residents of the Karamoja district	Cross-sectional, Purposive sampling	132 (49.2% M)	20-40 y, ave & IQR/SD NS	EBOV SUDV MARV	IFA	EBOV: 4 / 132, 3.0 (0.8-7.6)<br>SUDV: 4 / 132, 3.0 (0.8-7.6)<br>MARV: 6 / 132, 4.5 (1.7-9.6)
1993 (1961-62) Tignor, Ethiopia	General population: Adults and children in a yellow fever epidemic area and a non-epidemic area	Cross-sectional, Stratified sampling	277 (% M NS) *Non-epidemic area: 178* *Epidemic area: 99*	NS	EBOV MARV	IFA	EBOV: 54 / 277, 19.5 (15.0-24.7)<br>*Non-epidemic area: 42 / 178, 23.6 (17.6-30.5)*<br>*Epidemic area: 12 / 99, 12.1 (6.4-20.2)*<br><br>MARV: 0/277, 0.0 (0.0-1.3)<br>*Non-epidemic area: 0 / 178, 0.0 (0.0-2.1)*

Publication date (study date[a]) Author, Location	Study population – Population category[b]: Details	Study design, Sampling method[c]	Sample size (% M)	Participant age – Range, ave, IQR/SD	Virus evaluated [d]	Antibody detection assay[e]	Seroprevalence – N seropositive / total N, % (95% CI)[f]
							Epidemic area: 0 / 99, 0.0 (0.0-3.7)
2007 (2004) Onyango, South Sudan	Symptomatic / suspected cases: Suspected ED cases or close contacts	Outbreak investigation, Purposive sampling	17 (% M NS)	NS	'Ebola'[h] (Tested both EBOV & SUDV)	IgG ELISA<br>IgM ELISA	EBOV / SUDV: 12 / 17, 70.6 (44.0-89.7)
2011 (2007) Adjemian, Uganda	Close contacts: Contacts of confirmed MD cases identified through contact tracing	Outbreak investigation, Purposive sampling	83 (% M NS)	NS	MARV	IgG ELISA<br>IgM ELISA	0 / 83, 0.0 (0.0-4.3)
2015 (2008) Clark, Uganda	Close contacts: Persons identified as having close contact with a BDBV-positive case during the 2007 outbreak in Bundibugyo	Cohort study, Purposive sampling	223 (48.0% M)	25-40y, mean 33.9y, IQR NS	BDBV<br>EBOV<br>SUDV<br>TAFV<br>MARV	IgG ELISA	BDBV: 8 / 223, 3.6 (1.6-6.9)<br>EBOV: 11 / 223, 4.9 (2.5-8.6)<br>SUDV: 3 / 223, 1.3 (0.3-3.9)<br>TAFV: 1 / 223, 0.4 (0.0-2.5)<br>MARV: 0 / 223, 0.0 (0.0-1.6)
2015 (2012) Knust, Uganda	Close contacts: Persons who had close contact with confirmed or probable MD cases	Cross-sectional, Purposive sampling	173 (% M NS)	Newborn to 64y	MARV	IgG ELISA<br>IgM ELISA	6 / 173, 3.5 (1.3-7.4)
2019 (2017) Nyakarahuka, Uganda	Close contacts: Close contacts of suspected index case (identified >21d post contact)	Outbreak investigation, Purposive sampling	36 (% M NS)	NS	MARV	IgG ELISA<br>IgM ELISA	1 / 36, 2.8 (0.1-14.5)
2020 (2015) Nyakarahuka, Uganda	Exp. to wildlife: Workers from bat-inhabited mines	Cross-sectional, Miners: Purposive & snowball sampling, Gen. popn.: Snowball or 2-stage cluster sampling	724 (54.1% M)<br>Miners: 161<br>Gen. popn.: 563	3-82y, median 33y, IQR NS	EBOV<br>SUDV<br>BDBV<br>MARV	IgG ELISA	EBOV: 0 / 161, 0.0 (0.0-2.3)<br>SUDV: 8 / 161, 5.0 (2.2-9.6)<br>BDBV: 0 / 161, 0.0 (0.0-2.3)<br>MARV: 1 / 161, 0.6 (0.0-3.4)
	General population: Residents of households local to mines (excl. mine workers) and residents of unforested region away from mines						EBOV: 0 / 563, 0.0 (0.0-0.7)<br>SUDV: 10 / 563, 1.8 (0.9-3.2)<br>BDBV: 1 / 563, 0.2 (0.0-1.0)<br>MARV: 0 / 563, 0.0 (0.0-0.7)
2021 (2018) Rugarabamu, Tanzania	Symptomatic / suspected cases: Health facility outpatients	Cross-sectional, Cluster sampling	500 (41.8% M)<br>Symptomatic: 259<br>Gen. popn.: 241	Range NS (all ≥9m), mean 35y, SD 18.9y	EBOV<br>MARV	IgG ELISA<br>IgM ELISA	EBOV IgG: 7 / 259, 2.7 (1.1-5.5)<br>EBOV IgM: 4 / 259, 1.5 (0.4-3.9)<br>MARV IgG: 1 / 259, 0.4 (0.0-2.1)<br>MARV IgM: 1 / 259, 0.4 (0.0-2.1)

Publication date (study date[a]) Author, Location	Study population – Population category[b]: Details	Study design, Sampling method[c]	Sample size (% M)	Participant age – Range, ave, IQR/SD	Virus evaluated [d]	Antibody detection assay[e]	Seroprevalence – N seropositive / total N, % (95% CI)[f]
	General population: Community-level households						EBOV IgG: 2 / 241, 0.8 (0.1-3.0)<br>EBOV IgM: 4 / 241, 1.7 (0.5-4.2)<br>MARV IgG: 2 / 241, 0.8 (0.1-3.0)<br>MARV IgM: 2 / 241, 0.8 (0.1-3.0)
2022 (2018) Rugarabamu, Tanzania	Symptomatic / suspected cases: Febrile patients at primary HCF	Cross-sectional, Purposive sampling	308 (39.9% M)	NS	EBOV<br>MARV	IgM ELISA	EBOV: 3 / 308, 1.0 (0.2-2.8)<br>≤45y: 0 / 216, 0.0 (0.0-1.7)<br>46-60y: 3 / 55, 5.5 (1.1-15.1)<br>≥61y: 0 / 37, 0.0 (0.0-9.5)<br>MARV: 1 / 308, 0.3 (0.0-1.85)<br>0-15y: 1 / 49, 2.0 (0.1-10.9)<br>≥16y: 0 / 259, 0.0 (0.0-1.4)
**CENTRAL AFRICA**							
1978 (1976) Intl. Comm., DRC	Symptomatic / suspected cases: Ill persons	Cross-sectional, Stratified sampling	967 (% M NS)<br>Symptomatic: 121<br>Contacts: 404<br>Gen. popn.: 442	8-48 y in gen. popn., ave & IQR/SD NS	'Ebola'[g] (assumed to be EBOV, since study in DRC)	IFA	20 / 121, 16.5 (10.4-24.4)
	Close contacts: Individuals in close contact with Ebola-confirmed case						10 / 404, 2.5 (1.2-4.5)
	General population: Persons from villages with no documented Ebola cases						5 / 442, 1.1 (0.4-2.6)
1979 (1972-78) Van der Groen, DRC	General population: Populations of many different villages in northwestern DRC	Cross-sectional, Cluster sampling	251 (51.4% M)	0-≥40 y, ave & IQR/SD NS	'Ebola'[g] (assumed to be EBOV, since study in DRC)	IFA	43 / 251, 17.1 (12.7-22.4)
1980 (1977-78) Heymann, DRC	General population: Villages in the Tandala region of DRC, where an EBOV case had been documented in 1977	Cross-sectional, Cluster sampling	1096 (45.3% M)	≤4->60 y, ave & IQR/SD NS	'Ebola'[g] (assumed to be EBOV, since study in DRC)	Indirect IFA	79 / 1096, 7.2 (5.7-8.9)
1980 (1979) Saluzzo, CAR	General population: Residents of forested regions of the CAR	Cross-sectional, Cluster sampling	499 (55.9% M)	0-≥40 y, ave & IQR/SD NS	'Ebola'[g] (assumed to be	Indirect IFA	17 / 499, 3.4 (2.0-5.4)<br>0-9 y: 0 / 93, 0.0 (0.0-3.9)<br>10-19 y: 3 / 75, 4.0 (0.8-11.1)

Publication date (study date[a]) Author, Location	Study population – Population category[b]: Details	Study design, Sampling method[c]	Sample size (% M)	Participant age – Range, ave, IQR/SD	Virus evaluated [d]	Antibody detection assay[e]	Seroprevalence – N seropositive / total N, % (95% CI)[f]
					EBOV, since study in CAR)		20-29 y: 0 / 50, 0.0 (0.0-7.1)<br>30-39 y: 4 / 86, 4.7 (1.3-11.5)<br>≥40 y: 10 / 195, 5.1 (2.5-9.2)
1982 (1980) Ivanoff, Gabon	General population: Adults and newborns in Gabon	Cross-sectional, Convenience sampling	253 (% M NS)	Newborns and adults	EBOV<br>SUDV<br>MARV	IFA	EBOV: 19 / 253, 7.5 (4.6-11.5)<br>Adults: 18 / 197, 9.1 (5.5-14.1)<br>Newborns: 1 / 28, 3.6 (0.1-1.8)<br>SUDV: 5 / 253, 2.0 (0.6-4.6)<br>Adults: 5 / 197, 2.5 (0.8-5.8)<br>Newborns: 0 / 28, 0.0 (0.0-1.2)<br>MARV: 0 / 253. 0.0 (0.0-1.5)<br>Adults: 0 / 197, 0.0 (0.0-1.9)<br>Newborns: 0 / 28, 0.0 (0.0-1.2)
1982 (1981) Talani, ROC	General population: Children not vaccinated against smallpox, from various regions in the Pool region	Cross-sectional, Cluster sampling	790 (% M NS)	3-15 y, ave & IQR/SD NS	'Ebola'[g] (assumed to be EBOV, since study in Cameroon)<br>MARV	IFA	EBOV: 119 / 790, 15.1 (12.6-17.8)<br>MARV: 26 / 790, 3.3 (2.2-4.8)
1983 (1980) Bouree, Cameroon	Asymptomatic, healthy: Apparently healthy persons in five regions of Cameroon	Cross-sectional, Cluster sampling	1517 (% M NS)	0-≥30 y, ave & IQR/SD NS	'Ebola'[g] (assumed to be EBOV, since study in Cameroon)<br>MARV	Indirect IFA	EBOV: 147 / 1517, 9.7 (8.2-11.3)<br>0-5 y: 6 / 174, 3.4 (1.3-7.4)<br>6-20 y: 47 / 505, 9.3 (6.9-12.2)<br>21-30 y: 34 / 292, 11.6 (8.2-15.9)<br>>30 y: 60 / 546, 11.0 (8.5-13.9)<br>MARV: 7 / 1517, 0.5 (0.2-0.9)
1987a (1984-85) Meunier, CAR	General population: Residents of various different regions in the CAR	Cross-sectional, Cluster sampling	1528 (% M NS)	NS	EBOV<br>SUDV<br>MARV<br>*Only about half of samples were tested*	IFA	EBOV: 319 / 1528, 20.9 (19.9-23.0)<br>SUDV: 63 / 836, 7.5 (5.8-9.5)<br>MARV: 11 / 836, 1.3 (0.7-2.3)

Publication date (study date[a]) Author, Location	Study population – Population category[b]: Details	Study design, Sampling method[c]	Sample size (% M)	Participant age – Range, ave, IQR/SD	Virus evaluated [d]	Antibody detection assay[e]	Seroprevalence – N seropositive / total N, % (95% CI)[f]
					for SUDV and MARV		
1987b (1985) Meunier, Gabon	General population: Inhabitants of the village of Ambinda	Cross-sectional, Purposive sampling	213 (42% M)	NS	EBOV<br>SUDV<br>MARV	IFA	EBOV: 20 / 213, 9.4 (5.8-14.1)<br>SUDV: 2 / 213, 0.9 (0.1-0.3)<br>MARV: 0 / 213, 0.0 (0.0-1.7)
1988 (1985) Paix, Cameroon	General population: Urban population	Cross-sectional, Cluster sampling	375 (% M NS)	15-44y, ave & IQR/SD NS	EBOV<br>MARV	IFA	EBOV: 7 / 375, 1.9 (0.8-3.8)<br>MARV: 0 / 375, 0.0 (0.0-1.0)
1989 (1985-87) Gonzalez, CAR, Cameroon, Chad, ROC, Gabon, Equatorial Guinea	General population: Residents from rural & urban regions (ranging from dry savannah to rainforest) of 6 central African countries	Cross-sectional, Cluster sampling	5070 (% M NS)	NS	'Ebola'[h] (Tested both EBOV & SUDV)<br>MARV	IFA	EBOV / SUDV: 629 / 5070, 12.4 (11.5-13.3)<br>*Results not provided by specific virus*<br>Cameroon: 89 / 1152, 7.7 (6.3-9.4)<br>CAR: 107 / 327, 32.8 (27.7-38.1)<br>Chad: 12 / 334, 3.6 (1.9-6.2)<br>Congo: 51 / 728, 7.0 (5.3-9.1)<br>Eq. Guinea: 111 / 688, 16.1 (13.5-19.1)<br>Gabon: 259 / 1841, 14.1 (12.5-15.7)<br>MARV: 20 / 5070, 0.4 (0.2-0.6)<br>Cameroon: 0 / 1152, 0.0 (0.0-0.3)<br>CAR: 0 / 327, 0.0 (0.0-1.1)<br>Chad: 1 / 334, 0.3 (0.0-1.7)<br>Congo: 11 / 728, 1.5 (0.8-2.7)<br>Eq. Guinea: 8 / 688, 1.2 (0.5-2.3)<br>Gabon: 0 / 1841, 0.0 (0.0-0.2)
1993a (NS) Johnson, CAR	General population: Residents from 5 ecologically distinct zones (incl. grassland & forest); villages selected on basis of accessibility	Cross-sectional, NS	4295 (% M NS)	NS	EBOV<br>SUDV<br>MARV	IFA	EBOV: 681 / 4295, 15.9 (14.8-17.0)<br>SUDV: 853 / 4295, 19.9 (18.7-21.1)<br>MARV: 137 / 4295, 3.2 (2.7-3.8)
1993b (1987) Johnson, CAR	Exp. to wildlife: Hunter-gatherers and subsistence farmers living in forest environments	Cross-sectional, Convenience sampling	427 (% M NS)	NS	EBOV<br>SUDV<br>MARV	IFA	EBOV: 51 / 427, 11.9 (9.0-15.4)<br>SUDV: 68 / 427, 15.9 (12.6-19.7)<br>MARV: 4 / 427, 0.9 (0.3-2.4)
1999 (1996) Bertherat, Gabon	General population: Gold-panners & other community members from gold-panning villages previously affected	Cross-sectional, NS	236 (% M NS)	4-73y, mean 34y, SD 12y	EBOV	IgG ELISA<br>IgM ELISA	IgG: 24 / 236, 10.2 (6.6-14.8)<br>IgM: 0 / 236, 0.0 (0.0-1.6)

Publication date (study date[a]) Author, Location	Study population – Population category[b]: Details	Study design, Sampling method[c]	Sample size (% M)	Participant age – Range, ave, IQR/SD	Virus evaluated [d]	Antibody detection assay[e]	Seroprevalence – N seropositive / total N, % (95% CI)[f]
	by EBOV						
1999 (1995) Busico, DRC	Exp. to wildlife: Forest workers from forested surrounding Kikwit, where 1995 EBOV outbreak occurred	Cross-sectional/case series, Volunteer response sampling	575 (% M NS) Exp. wildlife: 230 Gen. popn.1: 184 Gen. popn.2: 161	Range NS, median of groups 1 & 2: 32y, median of group 3: 41y, IQR NS	EBOV	IgG ELISA	5 / 230, 2.2 (0.7-5.0)
	General population: City workers from Kikwit						4 / 184, 2.2 (0.6-5.5)
	General population: General population from villages nearby Kikwit with no reported ED cases						15 / 161, 9.3 (5.3-14.9)
1999 (1995) Tomori, DRC	HCW: Individuals working in a healthcare center or hospital in Kikwit or surrounding towns	Cross-sectional, Purposive sampling	402 (58% M)	NS	EBOV	IgG ELISA IgM ELISA	12 / 402, 3.0 (1.6-5.2)
1999 (1981-85) Jezek, DRC	Symptomatic / suspected cases: Suspected cases identified through surveillance of area previously affected by EBOV	Hemorrhagic fever surveillance, Purposive sampling	355 (% M NS) Symptomatic: 30 Contacts: 188 Asymptomatic: 137	NS	'Ebola'[h] (Tested both EBOV & SUDV)	IFA	EBOV / SUDV: 18 / 30, 60.0 (40.6-77.3)
	Close contacts: Household contacts of suspect cases and non-household contacts that visited or cared for suspected cases						EBOV / SUDV: 28 / 188, 14.9 (10.1-20.8)
	Asymptomatic, healthy: Age & sex matched controls living in same villages as suspected cases but with no reported contact						EBOV / SUDV: 2 / 137, 1.5 (0.2-5.2)
1999 (1995) Rowe, DRC	Close contacts: Household contacts of individuals with confirmed ED	Cohort study, Purposive sampling	101 (51.3% M)	3 months-58y, median 15y, IQR NS	EBOV	IgG ELISA IgM ELISA	4 / 101, 4.0 (1.1-9.8)
1999 (1994-95) Georges, Gabon	Close contacts: Contacts of sick persons who had	Cross-sectional, Purposive sampling	441 (% M NS) Contacts: 236	NS	EBOV	IFA IgG ELISA	24 / 236, 10.2 (6.6-14.8)

Publication date (study date[a]) Author, Location	Study population – Population category[b]: Details	Study design, Sampling method[c]	Sample size (% M)	Participant age – Range, ave, IQR/SD	Virus evaluated [d]	Antibody detection assay[e]	Seroprevalence – N seropositive / total N, % (95% CI)[f]
	hemorrhagic fever symptoms		Gen. popn.: 205			IgM ELISA	
	General population: Residents of Mayibout 2 and neighboring villages						34 / 205, 16.6 (11.8-22.4)
2000 (1994-1997) Nakounné, CAR	Exp. to wildlife: Pygmy & Bantu populations living in forested areas; hunters & gatherers	Cross-sectional, Purposive sampling	1778 (% M NS) Exp. to wildlife: 684 Gen. pop.: 1097	NS	EBOV MARV	IgG ELISA IFA	EBOV: 48 / 684, 7.0 MARV: 14 / 681, 2.1
	General population: Local Bantu villager populations						EBOV: 49 / 1091, 4.4 MARV: 18 / 1097, 1.6
2000 (1992-1996) Gonzalez, CAR	Exp. to wildlife: Farmers and Pygmies (hunter-gatherers) in remote forest areas	Cross-sectional, NS	1340 (% M NS)	NS	EBOV MARV	IgG ELISA	EBOV: 71 / 1331, 5.3 (4.2-6.7) MARV: 33 / 1340, 2.5 (1.7-3.4)
2000 (1996) Leroy, Gabon	Close contacts: Asymptomatic close contacts of symptomatic patients with laboratory-confirmed infections	Cohort study, Purposive sampling	24 (% M NS)	NS	EBOV	IgG ELISA IgM ELISA	11 / 24, 45.8 (25.6-67.2)
2003 (1999) Bausch, DRC	General population: General population of Durba, where 1999 MD outbreak occurred	Cross-sectional, Convenience sampling	912 (65.1% M)	14-79y, median 31y, IQR NS	MARV	IgG ELISA IgM ELISA *IgG IFA*	15 / 912, 1.6 (0.9-2.7)
	HCW: HCWs at all HCFs in Durba and larger nearby town (Watsa)		103 (% M NS)	NS			0 / 103, 0.0 (0.0-3.5)
2005 (NS) Borchert, DRC	Exp. to wildlife: Watsa pygmies - hunter-gatherer populations living seminomadically in forest	Cross-sectional, Voluntary response sampling	300 (50.0% M)	10-75y, median 30y, IQR NS	MARV	IgG ELISA IgG IFA	0 / 300, 0.0 (0.0-1.2)
2005 (1997) Heffernan, Gabon	General population: Residents from 8 villages, incl. site of 1996 ED outbreak	Cross-sectional, NS	975 (48.5% M)	NS	EBOV	IgG ELISA	10 / 975, 1.0 (0.5-1.9)
2006 (2002) Borchert, DRC	Close contacts: Household contacts of confirmed MD cases	Cross-sectional, Purposive sampling	121 (52.1% M)	NS	MARV	IgM ELISA IgG IFA	2 / 121, 1.7 (0.2-5.8)
2006 (2003) Formenty, ROC	Symptomatic / suspected cases: Patients meeting case	Cross-sectional, Purposive sampling	34 (% M NS) Symptomatic: 24	28-75y, ave & IQR/SD NS	EBOV	IgG ELISA	6 / 24, 25.0 (9.8-46.7)

Publication date (study date[a]) Author, Location	Study population – Population category[b]: Details	Study design, Sampling method[c]	Sample size (% M)	Participant age – Range, ave, IQR/SD	Virus evaluated[d]	Antibody detection assay[e]	Seroprevalence – N seropositive / total N, % (95% CI)[f]
	definition for ED		Asymptomatic: 10				
	Asymptomatic, healthy: Healthy volunteers						0 / 10, 0.0 (0.0-30.8)
2007 (1981-97) Lahm, Gabon	General population: Residents of various rural communities in Gabon	Cross-sectional, Cluster sampling	1147 (% M NS)	10-75 y, ave & IQR/SD NS	EBOV	IgG ELISA<br>IgM ELISA	14 / 1147, 1.2 (0.7-2.0)<br>*IgM ELISA: 0 / 1147, 0.0 (0.0, 0.3)*
2007 (2001-02) Borchert, DRC	HCW: HCW who cared for confirmed MD cases, or who had contact with body fluids	Cross-sectional, Purposive sampling	48 (70.8% M)	23-62y, median 34y, IQR NS	MARV	IgG ELISA<br>IgG IFA	1 / 48, 2.1 (0.1-11.1)
2010 (2005-08) Becquart, Gabon	Asymptomatic, healthy: Healthy rural adult Gabonese populations from 220 villages	Cross-sectional, Stratified random sampling	4349 (48.4% M)	16-65y, ave & IQR/SD NS	EBOV	IgG ELISA	667 / 4349, 15.3 (14.3-16.4)
2011 (2005-08) Nkoghe, Gabon[i]	Asymptomatic, healthy: Healthy rural adult Gabonese populations from 220 villages	Cross-sectional, Stratified random sampling	4349 (47.1% M)	16-90y, mean 46y, SD 14y	EBOV	IgG ELISA	667 / 4349, 15.3 (14.3-16.4)
	Asymptomatic, healthy: Healthy rural child Gabonese populations from 6 villages		362 (56.2% M)	1-15y, ave & IQR/SD NS			47 / 362, 13.0 (9.7-16.9)
2015 (2011) Moyen, ROC	General population: Blood donors from urban & rural areas	Cross-sectional, NS	809 (75.2% M)	18-63y, mean 33y, SD 10.21y	EBOV<br>MARV	IgG IFA	EBOV: 20 / 809, 2.5 (1.5-3.8)<br>MARV: 4 / 809, 0.5 (0.1-1.3)
2016 (2002) Mulangu, DRC	Exp. to wildlife: Efé pygmies - hunter-gatherer populations living in tropical rainforests	Cross-sectional, Voluntary response sampling	300 (% M NS)	Range NS (all ≥10y), mean 32y, SD 14.6y	EBOV	IgG ELISA	56 / 300, 18.7 (14.4-23.5)<br>*Linear ↑ in prevalence with ↑ age group*
2017 (2014-15) Mbala, DRC	Close contacts: Asymptomatic residents of households with ≥1 confirmed or probable ED case during 2014 outbreak	Cross-sectional, Contacts: Purposive sampling, Gen. popn.: NS	370 (48.1% M)<br>Contacts: 182<br>Gen. popn.: 188	NS	EBOV	IgG ELISA<br>IgM ELISA	IgG: 2 / 182, 1.1 (0.1-3.9)<br>IgM: 1 / 182, 0.5 (0.0-3.0)
	General population: Households in area unaffected by 2014 ED outbreak						IgG: 3 / 188, 1.6 (0.3-4.6)<br>IgM: 1 / 182, 0.5 (0.0-3.0)

Publication date (study date[a]) Author, Location	Study population – Population category[b]: Details	Study design, Sampling method[c]	Sample size (% M)	Participant age – Range, ave, IQR/SD	Virus evaluated [d]	Antibody detection assay[e]	Seroprevalence – N seropositive / total N, % (95% CI)[f]
2018 (2007) Mulangu, DRC	Asymptomatic, healthy:: Healthy, rural village populations not previously affected by EBOV cases or outbreaks	Cross-sectional, Cluster sampling	3415 (42.1% M)	NS	EBOV	IgG ELISA	353 / 3331, 10.6 (9.6-11.7)<br>0-14y: 71 / 1407, 5.0 (4.0-6.3)<br>15-49y: 219 / 1562, 14.0 (12.3-15.8)<br>≥50y: 63 / 362, 17.4 (13.6-21.7)
2019 (2015) Hoff, DRC	HCW: Clinical & non-clinical HCW (incl. informal care givers*) actively involved in 2014 Boende outbreak response	Cross-sectional, Purposive sampling	565 (65.5% M)	Range NS (all >18y), median 40y, IQR 32-50y	EBOV	IgG ELISA<br>Luciferase IP<br>Neutralization assay	234 / 565, 41.4 (37.3-45.6)<br>Binding: 234 / 565, 41.4 (37.3-45.6)<br>Neutralizing: 16 / 565, 2.8 (1.6-4.6)
2020 (2018) Lucas, DRC	Exp. to wildlife: Bushmeat market vendors in province affected by ED outbreak	Cross-sectional, Stratified sampling	19 (0.0% M)	NS	EBOV<br>*SUDV*<br>*BDBV*<br>*TAFV*<br>*MARV*	IgG ELISA	1 / 19, 5.2 (0.1-26.0)<br>*EBOV +ve sample tested -ve for antibodies to SUDV, BDBV, TAFV & MARV*
2020 (2017-18) Goldstein, DRC	Symptomatic / suspected cases: Febrile patients seeking care in North Kivu (≤14m before documented start of ED outbreak)	Cross-sectional, Purposive sampling	272 (39.7% M)	2-68y, ave & IQR/SD NS	EBOV<br>*SUDV*<br>*BDBV*<br>*RESTV*<br>*MARV*	IgG ELISA	EBOV: 29 / 272, 10.7 (7.3-15.0)<br>Adults: 16 / 152, 10.5 (6.1-16.5)<br>Children: 13 / 120, 10.8 (5.9-17.8)<br>*EBOV +ve samples were tested for antibodies to SUDV, BDBV, RESTV, & MARV, and one sample tested +ve for BDBV*
2021 (2015-17) Bratcher, DRC	HCW: Clinical & non-clinical workers at HCF, & informal care givers	Cross-sectional, Convenience sampling	1,366 (62.6% M)<br>HCW: 959<br>Gen. popn.: 407	NS (all ≥18y)	EBOV	IgG ELISA	69 / 959, 7.2 (5.6-9.0)
	General population: Residents from semi-urban or rural regions						44 / 407, 10.8 (8.0-14.2)
2022 (2018) Nkuba-Ndaye, DRC	Symptomatic / suspected cases: Suspected ED patients with 2x consecutive -ve RT-PCR	Cross-sectional study, Symptomatic: SRS, Gen popn.: NS	488 (50.3% M)	Range & ave NS, IQR 12-32y	EBOV	IgG multiplex	11 / 488, 2.3 (1.1-4.0)
	General population: PLHIV undergoing ART monitoring pre ED outbreak		280 (31.0% M)	NS (all ≥18y)			1 / 280, 0.4 (0.0-2.0)
2022 (2018) Shaffer, DRC	HCW: Clinical & non-clinical workers at HCF; & informal care givers	Cross-sectional, Convenience sampling	539 (48.2% M)	Range & ave NS (all ≥18y), IQR 34-53y	EBOV<br>SUDV<br>BDBV	IgG ELISA	EBOV: 25 / 539, 4.6 (3.0-6.8)<br>SUDV: 12 / 539, 2.2 (1.2-3.9)<br>BDBV: 13 / 539, 2.4 (1.3-4.1)

Publication date<br>(study date[a])<br>Author, Location	Study population – Population category[b]: Details	Study design, Sampling method[c]	Sample size (% M)	Participant age – Range, ave, IQR/SD	Virus evaluated [d]	Antibody detection assay[e]	Seroprevalence –<br>N seropositive / total N, % (95% CI)[f]
2022 (2015)<br>Doshi, DRC	HCW: Clinical & non-clinical workers at HCF, & informal care givers	Cross-sectional, Purposive sampling	582 (64.4% M)	NS (all ≥18y)	EBOV	IgG ELISA	131 / 582, 22.5 (19.2-26.1)
2023 (2019-20)<br>Matuvanga, DRC	HCW: Healthy trial participants – healthcare providers & frontliners	Cross-sectional (nested in RCT), Convenience sampling	698 (76.5% M)	19-75y, ave & IQR/SD NS	EBOV	IgG ELISA<br>IgG multiplex	ELISA: 9 / 693, 7.1 (6.3-9.2)<br>Multiplex: 10 / 698, 1.4 (0.7–2.6)
**WEST AFRICA**							
1982 (1978-79)<br>Knobloch, Liberia	General population: Liberian citizens and non-Liberian nationals in rural areas of Liberia	Cross-sectional, Cluster sampling	433 (% M NS)	9 days-75y, ave & IQR/SD NS	'Ebola'[g] (assumed to be EBOV, since study in Liberia)<br>MARV	IFA	EBOV: 26 / 433, 6.0 (4.0-8.7)<br>MARV: 5 / 433, 1.2 (0.4-2.7)
1985 (1981-82)<br>Van der Waals, Liberia	Asymptomatic, healthy: Healthy relatives of epilepsy patients and unrelated, geographically matched controls	Cross-sectional, Purposive sampling	225 (% M NS)<br>Asymptomatic: 119<br>Symptomatic: 106	Mean 23.9y (± 2.5y) in asymptomatic individuals<br><br>Mean 16.7y (± 0.8y) in epilepsy patients<br>Range & IQR NS	EBOV<br>SUDV<br>MARV	IFA	EBOV: 15 / 119, 12.6 (7.2-19.9)<br>SUDV: 2 / 119, 1.7 (0.2-5.9)<br>MARV: 3 / 119, 2.5 (0.5-7.2)
	Symptomatic / suspected cases: Epilepsy patients inhabiting Grand Bassa County						EBOV: 11 / 106, 10.4 (5.3-17.8)<br>SUDV: 2 / 106, 1.9 (0.2-6.7)<br>MARV: 0 / 106, 0.0 (0.0-3.4)
1988 (1987)<br>Tomori, Nigeria	Asymptomatic, healthy: Apparently healthy individuals from various regions of Nigeria	Cross-sectional, Cluster sampling	1677 (56.6% M)	5-70y, ave & IQR/SD NS	SUDV<br>*EBOV*<br>MARV	IFA	SUDV: 30 / 1677, 1.8 (1.2-2.5)<br>*22 of 30 samples +ve for SUDV by IFA also neutralized EBOV*<br>MARV: 29 / 1677, 1.7 (1.2-2.5)
2014 (2006-08)<br>Schoepp, Sierra Leone, Liberia, Guinea	Symptomatic / suspected cases: Samples from acutely ill suspected LF patients submitted to Lassa Diagnostic Laboratory & -ve for malaria parasites & LASV	Cross-sectional, Purposive sampling	220 (% M NS)	NS	EBOV<br>*SUDV*<br>*TAFV*<br>MARV	IgG ELISA<br>IgM ELISA<br>Neutralization assay	EBOV: 19 / 220, 8.6 (5.3-13.2)<br>*14 of 18 samples +ve for IgM-only tested for neutralization. 8 neutralized EBOV, 1 SUDV, & 1 TAFV (but not considered evidence of TAFV infection). Seropositivity not reported by country*<br>MARV: 8 / 220, 3.6 (1.6-7.0)<br>*Seropositivity not reported by country*

Publication date (study date[a]) Author, Location	Study population – Population category[b]: Details	Study design, Sampling method[c]	Sample size (% M)	Participant age – Range, ave, IQR/SD	Virus evaluated [d]	Antibody detection assay[e]	Seroprevalence – N seropositive / total N, % (95% CI)[f]
2015 (2011-14) Boisen, Sierra Leone	Symptomatic / suspected cases: Samples from acutely ill suspected LF patients at Kenema General Hospital	Cross-sectional, Purposive sampling	242 (% M NS)	NS	EBOV	IgG ELISA<br>IgM ELISA	53 / 242, 21.9 (16.9-27.6)
2016 (2007-14) O'Hearn, Sierra Leone	Symptomatic / suspected cases: Samples from suspected LF patients & their contacts submitted to Lassa Diagnostic Laboratory	Cross-sectional, Purposive sampling	675 (% M NS)	NS	EBOV<br>MARV	IgG multiplex	EBOV: 35 / 672, 5.2 (3.6-7.2)<br>MARV: 71 / 663, 10.7 (8.5-13.3)
2016 (2015-16) Richardson, Sierra Leone	Close contacts: Minimally symptomatic quarantined contacts of confirmed ED cases	Cross-sectional, Purposive sampling	187 (57.8% M)	NS (all ≥4y)	EBOV	IgG ELISA	14 / 187, 7.5 (4.2-12.2)<br>*2 reported fever during quarantine period*
2016 (2009-13) Safronetz, Mali	Symptomatic / suspected cases: Acutely ill patients with history of fever and hemorrhagic, diarrheal, or icteric syndromes	Cross-sectional, Purposive sampling	376 (% M NS)	NS	EBOV	IgG ELISA<br>IgM ELISA	0 / 376, 0.0 (0.0-1.0)
2017 (2015-16) Houlihan, Sierra Leone, Liberia, Guinea	Close contacts: International HCW and lab responders during 2014-2016 ED outbreak	Cross-sectional, Snowball sampling	268 (43.3% M)<br>Contacts: 213<br>HCW: 55	≥18y, median 36y, IQR 31-40y	EBOV	IgG capture assay<br>*On oral swabs*	2 / 213, 0.9 (0.1-3.4)<br>*Seropositivity not reported by country*
	HCW: International responders, excl. HCW & lab personnel						0 / 55, 0.0 (0.0-6.5)<br>*Seropositivity not reported by country*
2017 (2015) Glynn, Sierra Leone	Close contacts: Household members of ED survivors from 2014-2015 outbreak	Cross-sectional, Contacts: Purposive sampling, Gen. popn.: Volunteer response sampling	820 (% M NS)<br>Contacts: 481<br>Gen. popn.: 339	NS	EBOV	IgG capture assay<br>*On oral swabs*	21 / 480, 4.4 (2.7-6.6)<br>Symptomatic: 11/ 92, 12.0 (6.1-20.4)<br><20y: 5 / 54, 9.3 (3.1-20.3)<br>≥20y: 6 / 38, 15.8 (6.0-31.3)<br>Asymptomatic: 10 / 388, 2.6 (1.2-4.7)<br><20y: 5 / 271, 1.8 (0.6-4.3)<br>≥20y: 5 / 117, 4.3 (1.4-9.7)
	General population: Community controls from areas with no known ED cases						0 / 339, 0.0 (0.0-0.1.1)

Publication date (study date[a]) Author, Location	Study population – Population category[b]: Details	Study design, Sampling method[c]	Sample size (% M)	Participant age – Range, ave, IQR/SD	Virus evaluated [d]	Antibody detection assay[e]	Seroprevalence – N seropositive / total N, % (95% CI)[f]
2017 (2015) Mafopa, Sierra Leone	Close contacts: Asymptomatic individuals from quarantined communities	Cross-sectional, Purposive sampling	184 (% M NS)<br>Contacts: 105<br>HCW: 79	NS	EBOV	IgG ELISA	12 / 105, 11.4 (6.0-19.1)
	HCW: Clinical & non-clinical workers from HCF not involved in management of ED cases						3 / 79, 3.8 (0.8-10.7)
2018 (2012) Keita, Guinea	General population: Demographic Health Survey participants nationwide prior to the 2014-2016 ED outbreak	Cross-sectional, DHS uses probability sampling; method for sampling from DHS NS	1483 (% M NS)	15-59y, ave & IQR/SD NS	EBOV	IgG multiplex	1 / 1483, 0.1 (0.0-0.4)
2018 (2015-16) Kelly, Sierra Leone	Close contacts: Residents of households quarantined in 2014-2015 during Ebola outbreak	Cross-sectional, Purposive sampling	221 (58.8% M)	NS	EBOV	IgG ELISA	48 / 221, 21.7 (16.5-27.7)<br>*Among 48 seropositive, 34 were confirmed ED cases, 2 were unreported ED cases, 12 were minimally symptomatic*<br><15y: 5 / 70, 7.1 (2.4-15.9)<br>15-45y: 33 / 125, 26.4 (18.9-35.0)<br>>45y: 10 / 26, 38.5 (20.2-59.4)
2019 (2017) Timothy, Guinea	General population: All households in village where EBOV spillover event occurred in 2013	Cross-sectional, Purposive sampling	237 (49.4% M)	Range NS (all ≥18y), mean 30y, SD NS	EBOV	IgG ELISA<br>*On oral swabs*	8 / 224, 3.6 (1.5-6.9)<br>*6 / 8 retrospectively considered suspected cases*
2019 (2016-17) Diallo, Guinea	Close contacts: Paucisymptomatic or asymptomatic close contacts of confirmed ED cases	Cross-sectional, Purposive sampling	1390<br>(49.1% M)	7-88y, median 26y, IQR NS	EBOV	IgG multiplex	57 / 1390, 4.1 (3.1-5.3)<br>Paucisymptomatic: 18 / 216, 8.3 (5.0-12.9)<br>Asymptomatic: 39 / 1174, 3.3 (2.4-4.5)<br>*↑ seroprevalence with ↑ age in paucisymptomatic contacts*
2019 (2017) Halfmann, Sierra Leone	Close contacts: Relatives & HCW who cared for ED patients	Cross-sectional, Purposive sampling	305 (% M NS)<br>Contacts: 267<br>Gen popn.: 38	NS	EBOV	IgG ELISA	107 / 267, 40.1 (34.1-46.2)<br>Relatives: 91 / 211, 43.1 (36.3-50.1)<br>HCW: 16 / 56, 28.6 (17.3-42.2)
	General population: People with no known exposure to EBOV						0 / 38, 0.0 (0.0-9.3)

**Publication date (study date[a]) Author, Location**	**Study population – Population category[b]: Details**	**Study design, Sampling method[c]**	**Sample size (% M)**	**Participant age – Range, ave, IQR/SD**	**Virus evaluated [d]**	**Antibody detection assay[e]**	**Seroprevalence – N seropositive / total N, % (95% CI)[f]**
2020 (2011-12) Surtees, Guinea	Symptomatic / suspected cases: Febrile patients	Cross-sectional, Purposive sampling	129 (% M NS)	NS	EBOV MARV	IgG multiplex	EBOV: 11 / 129, 8.5 (4.3-14.7)<br>MARV: 24 / 129, 18.6 (12.3-26.4)
2021 (2016-17) Dedkov, Guinea	Symptomatic / suspected cases: Patients hospitalized with acute febrile illness	Cross-sectional, Purposive sampling	164 (55.5% M)	2-75y, ave & IQR/SD NS	EBOV MARV	IgM microarray	EBOV: 0 / 164, 0.0 (0.0-2.2)<br>MARV: 0 / 164, 0.0 (0.0-2.2)
2021 (2015) Bane, Mali	Asymptomatic, healthy: Healthy volunteers	Cross-sectional, Voluntary response sampling	600 (% M NS)	NS	EBOV	IgG ELISA	37 / 600, 6.2 (4.4-8.4)
2022 (2016-18) Manno, Sierra Leone	Asymptomatic, healthy: Healthy trial participants	Cross-sectional (nested in RCT), Convenience sampling	1282 (64.5% M)	Range & ave NS (all ≥1y), IQR 7-25y	EBOV	IgG ELISA	107 / 1272, 8.4 (7.0-10.0)<br>1-4y: 14 / 240, 5.8 (3.2-9.6)<br>5-9y: 18 / 168, 10.7 (6.5-16.4)<br>10-19y: 24 / 353, 6.8 (4.4-9.9)<br>20-39y: 39 / 387, 10.1 (7.3-13.5)<br>≥40y: 12 / 124, 9.7 (5.1-16.3)
2022a (2016-17) Kelly, Sierra Leone	Close contacts: Household contacts of ED cases	Cross-sectional, Purposive sampling	421 (58.0% M)	≤19-≥50y	EBOV	IgG ELISA	40 / 421, 9.5 (6.9-12.7)<br>*18 / 40 (45.0%) symptomatic*
2022b (2015-17) Kelly, Liberia	Close contacts: Contacts of ED survivors	Longitudinal cohort study, Purposive sampling	2688 (44.1% M)	Range & ave NS, IQR 15-36y	EBOV	IgG ELISA	202 / 2688, 8.3 (7.2-9.4)<br>*107 / 222 (48.2%) symptomatic*
2023 (2015-17) Gayedyu-Dennis, Liberia[j]	Close contacts: Contacts of ED survivors	Longitudinal cohort study, Purposive sampling	1909 (44.3% M)	≤10-≥41y	EBOV	IgG ELISA	222 / 1909, 12.0 (10.2-13.2)<br>*115 / 222 (51.8%) paucisymptomatic or asymptomatic*<br>*107 / 222 (48.2%) with unrecognized, symptomatic ED*
**SOUTHERN AFRICA**							
1978 (1975) Conrad, Zimbabwe	Close contacts: People who might have had contact with 2 confirmed MD cases before they became symptomatic, or with other seriously ill people / animals	Outbreak investigation, Purposive sampling	234 (% M NS)	NS	MARV	IFA	0 / 234, 0.0 (0.0-1.6)
1982 (1980) Blackburn, Zimbabwe	General population: Schoolchildren from four communal areas in Zimbabwe	Cross-sectional, Purposive sampling	486 (% M NS)	8-10 y, ave & IQR/SD NS	EBOV *SUDV* MARV	IFA	EBOV: 9 / 486, 1.9 (0.9-3.5)<br>*EBOV +ve samples were tested for antibodies to SUDV, and no samples tested +ve for SUDV*

Publication date (study date[a]) Author, Location	Study population – Population category[b]: Details	Study design, Sampling method[c]	Sample size (% M)	Participant age – Range, ave, IQR/SD	Virus evaluated[d]	Antibody detection assay[e]	Seroprevalence – N seropositive / total N, % (95% CI)[f]
							MARV: 4 / 486, 0.8 (0.2-2.1)
1987 (1984-86) Tessier, Botswana	Exp. to wildlife: Villagers with documented wildlife hunting activities	Cross-sectional, Stratified sampling	154 (% M NS)<br>Exp. to wildlife: 52<br>Symptomatic: 25<br>Gen. popn.: 77	18 months-80 y, ave & IQR/SD NS	EBOV<br>SUDV<br>MARV	IFA	EBOV: 0 / 52, 0.0 (0.0-6.8)<br>SUDV: 0 / 52, 0.0 (0.0-6.8)<br>MARV: 0 / 52, 0.0 (0.0-6.8)
	Symptomatic / suspected cases: Patients with non-specific serious symptoms or hemorrhagic syndromes						EBOV: 0 / 25, 0.0 (0.0-13.7)<br>SUDV: 0 / 25, 0.0 (0.0-13.7)<br>MARV: 0 / 25, 0.0 (0.0-13.7)
	General population: Villagers from Chobe region with no reported symptoms or exposures						EBOV: 0 / 77, 0.0 (0.0-4.7)<br>SUDV: 0 / 77, 0.0 (0.0-4.7)<br>MARV: 0 / 77, 0.0 (0.0-4.7)
1989 (1976) Mathiot, Madagascar	Asymptomatic, healthy: Apparently healthy adults from various regions in Madagascar	Cross-sectional, Cluster sampling	381 (% M NS)	NS	EBOV<br>SUDV<br>MARV	IFA	EBOV: 17 / 381, 4.5 (2.6-7.1)<br>SUDV: 0 / 381, 0.0 (0.0-1.0)<br>MARV: 0 / 381, 0.0 (0.0-1.0)
**MULTI-REGION**							
1984 (1984) Slenczka, Sierra Leone, South Sudan	General population: Samples from Sierra Leone, South Sudan, and without specific designations	Cross-sectional, Cluster sampling	1108 (% M NS)<br>Sierra Leone: 556<br>South Sudan: 284<br>Undesignated location: 268	NS	'Ebola'[g] (assumed EBOV in Sierra Leone, SUDV in Sudan)<br>MARV	IgG enzyme immunoassay<br>IFA	EBOV: 20 / 556, 3.6 (2.2-5.5)<br>SUDV: 1 / 284, 0.4 (0.0-1.9)<br>MARV: 0 / 1108, 0.0 (0.0-0.3)
2019 (various 1997-2012) Steffen, Uganda, DRC, ROC, Cameroon, Ghana	Symptomatic / suspected cases: Patients with illness of unknown etiology (Cameroon)	Cross-sectional, NS	2430 (% M NS)<br>Symptomatic: 160<br>Gen. popn.: 2270	NS	EBOV	ELISA<br>Neutralization assay<br>Luciferase IP	2 / 160, 1.3 (0.2-4.4)<br>*Seropositivity not reported by country*
	General population: PLHIV (Uganda, Cameroon, Ghana, ROC), blood donors (DRC), & monkeypox surveillance population (DRC)						51 / 2270, 2.2 (1.7-2.9)<br>Uganda: 0 / 106, 0.0 (0.0-3.4)<br>Cameroon: 0 / 96, 0.0 (0.0-3.8)<br>ROC: 9 / 458, 2.0 (0.9-3.7)<br>DRC: 42 / 1562, 2.7 (1.9-3.6)<br>Ghana: 0 / 48, 0.0 (0.0-7.4)

Publication date (study date[a]) Author, Location	Study population – Population category[b]: Details	Study design, Sampling method[c]	Sample size (% M)	Participant age – Range, ave, IQR/SD	Virus evaluated[d]	Antibody detection assay[e]	Seroprevalence – N seropositive / total N, % (95% CI)[f]
2020 (various 1997-2012) Steffen, Uganda, DRC, ROC, Cameroon, Ghana	Symptomatic / suspected cases: Patients with illness of unknown etiology (Cameroon)	Cross-sectional, NS	2430 (% M NS) Symptomatic: 160 Gen. popn.: 2270	NS	MARV	Neutralization assay *IgG ELISA*	12 / 160, 7.5 (3.9-12.7) *Seropositivity not reported by country*
	General population: PLHIV (Uganda, Cameroon, Ghana, ROC), blood donors (DRC), & monkeypox surveillance population (DRC)						14 / 2270, 0.6 (0.3-1.0)<br>Uganda: 3 / 106, 2.8 (0.6-8.0)<br>Cameroon: 0 / 96, 0.0 (0.0-3.8)<br>ROC: 4 / 458, 0.9 (0.2-2.2)<br>DRC: 4 / 1562, 0.3 (0.1-0.7)<br>Ghana: 3 / 48, 6.3 (1.3-17.2)

Abbreviations: ART, antiretroviral therapy; ave, average; BDBV, Bundibugyo virus; CAR, Central African Republic; CI, confidence interval; d, days; DBS, dried blood spot; DHS, demographic health survey; DRC, Democratic Republic of Congo; EBOV, Ebolavirus; ELISA, enzyme-linked immunosorbent assay; ED, Ebola disease; Exp., exposed; Gen. popn., general population; HCF, healthcare facility; HCW, healthcare worker(s); IFA, immunofluorescence assay; IgG, immunoglobulin G; IgM, immunoglobulin M; IQR, interquartile range; LF, Lassa fever; M, male; MARV, Marburgvirus; MD, Marburg disease; m, months; N, number; NS, not stated; PLHIV, people living with HIV; RCT, randomized controlled trial; RESTV, Reston virus; ROC, Republic of Congo; RT-PCR, reverse transcriptase polymerase chain reaction; SD, standard deviation; SRS, simple random sampling; SUDV, Sudan virus; TAFV, Taï Forest virus; VHF, viral hemorrhagic fever; y, years; +ve, positive; -ve, negative.

[a] Or date of sample collection if earlier.

[b] Hypothetical risk of viral infection, including: (1) 'symptomatic / suspected cases' (considered at high risk); (2) 'close contacts' of suspected, probable or confirmed ED or MD cases (considered at high risk); (3) 'HCW', inclusive of clinical and non-clinical workers at HCF, and informal care givers such as traditional healers and pastors (considered at moderate risk); (4) populations with 'exposure to wildlife' (considered at moderate risk); (5) 'general populations' (considered at low risk); and (6) 'asymptomatic / healthy' populations (considered at low risk). Where participant groups overlapped multiple categories, the category with the highest risk was assigned.

[c] Refers to methods for selection of study participants.

[d] Viruses written in italics were only evaluated among participants who tested EBOV antibody seropositive.

[e] All assays used blood components (serum, plasma or DBS) unless stated otherwise. Details of samples, assays and cutoffs / definitions used are provided in Supplementary Table 6. Assays written in italics were only used for samples where the primary assay yielded a positive result.

[f] To standardize reporting, seroprevalence and 95% CIs were calculated by the authors of this review using numerator and denominator data extracted from the included articles. 95% CI were calculated using the Clopper-Pearson (Exact).

[g] These articles did not provide details of the antigen used in the assay and just described measurement of antibodies to 'Ebola,' so based on the documented Ebola species endemicity of the country at the time during which these studies were conducted, we classified each study as either EBOV (Central or West Africa) or SUDV (East Africa).
[h] These studies used an assay that measures antibody responses to EBOV and SUDV simultaneously, and therefore did not present results for each specific virus.
[i] This article describes the same study as the article listed above (Becquart, 2010).
[j] This article describes the same study as the article listed above (Kelly, 2022b).

Table S5: Risk of bias assessment and GRADE rating for studies measuring antibody seroprevalence.

Year[a], Author, Location	Appropriateness / adequacy of: [b, c]									Risk of Bias Rating	GRADE Rating
	Sample frame	Participant sampling	Sample size	Description of setting & participants	Sample coverage for data analysis	Methods / measurements (validity)	Methods / measurements (standardized, reliable)	Statistical analysis	Response rate		
**EAST AFRICA**											
1982, Johnson, Kenya	No	Unclear	Yes	No	Maybe	Unclear	Maybe	No	Maybe	Moderate	Moderate
1983, Baron, Sudan	Unclear	Yes	Unclear	Yes	Maybe	Unclear	Maybe	No	Yes	Low	Moderate
1982, Smith, Kenya	Yes	Yes	Yes	No	Maybe	No	No	No	Maybe	Moderate	Low
1983, Johnson, Kenya	Unclear	Unclear	Yes	No	Maybe	No	No	No	Maybe	Moderate	Low
1989, Rodhain, Uganda	Yes	Unclear	Unclear	Maybe	Maybe	No	No	No	Maybe	Moderate	Moderate
1993, Tignor, Ethiopia	Yes	Unclear	Yes	No	Maybe	No	No	No	Maybe	Moderate	Low
2007, Onyango, Sudan	Yes	Yes	No	Maybe	Maybe	Yes	Maybe	No	Maybe	Moderate	Low
2011, Adjemian, Uganda	Yes	Unclear	Unclear	Maybe	No	Yes	Maybe	No	Maybe	Moderate	Moderate
2015, Clark, Uganda	Yes	Yes	Yes	Yes	Yes	Yes	Yes	No	Yes	Very Low	High
2015, Knust, Uganda	Unclear	Yes	Yes	No	No	Yes	Yes	No	Yes	Low	Moderate
2019, Nyakarahuka, Uganda	Yes	Yes	No	No	Maybe	Yes	Yes	No	Maybe	Low	Moderate
2020, Nyakarahuka, Uganda	Maybe	Yes	Yes	Yes	Maybe	Yes	Yes	Maybe	Maybe	Low	High
2021, Rugarabamu, Tanzania	Yes	Yes	Yes	Yes	Yes	Yes	Yes	Yes	Yes	Very Low	High
2022, Rugarabamu, Tanzania	Yes	Yes	Yes	Yes	Maybe	Unclear	Yes	Yes	Maybe	Low	Moderate
**CENTRAL AFRICA**											
1978, Intl. Comm., DRC	Yes	Yes	Yes	No	Maybe	No	No	No	Maybe	Moderate	Low
1979, Van der Groen, DRC	Unclear	Unclear	Yes	Yes	Maybe	No	No	No	Maybe	Moderate	Low
1980, Heymann, DRC	Yes	Unclear	Yes	Yes	Maybe	No	No	No	Maybe	Moderate	Low
1980, Saluzzo, CAR	Yes	Unclear	Yes	No	Maybe	No	No	No	Maybe	Moderate	Low

Year[a], Author, Location	Appropriateness / adequacy of: [b, c]									Risk of Bias Rating	GRADE Rating
	Sample frame	Participant sampling	Sample size	Description of setting & participants	Sample coverage for data analysis	Methods / measurements (validity)	Methods / measurements (standardized, reliable)	Statistical analysis	Response rate		
1982, Ivanoff, Gabon	Unclear	Unclear	Yes	No	Maybe	No	Yes	No	Maybe	Moderate	Low
1982, Talani, ROC	Yes	Unclear	Yes	No	Maybe	No	No	No	Maybe	Moderate	Low
1983, Bouree, Cameroon	Yes	Unclear	Yes	No	Maybe	No	No	No	Maybe	Moderate	Low
1987a, Meunier, CAR	Unclear	Unclear	Yes	No	Maybe	No	No	No	Maybe	Moderate	Low
1987b, Meunier, Gabon	Unclear	Unclear	Yes	Maybe	Maybe	Unclear	Maybe	No	Maybe	Moderate	Moderate
1988, Paix, Cameroon	Unclear	Unclear	Yes	No	Maybe	Unclear	Maybe	No	Maybe	Moderate	Low
1989, Gonzalez, Multi	Unclear	Yes	Yes	No	Maybe	Unclear	Maybe	Maybe	Maybe	Low	Low
1993a, Johnson, CAR	Unclear	Maybe	Yes	Maybe	Maybe	No	No	Maybe	Maybe	Moderate	Low
1993b, Johnson, CAR	Unclear	Maybe	Yes	Maybe	Maybe	Maybe	Maybe	Maybe	Maybe	Moderate	Low
1999, Busico, DRC	Unclear	Yes	Yes	Yes	Maybe	Yes	Yes	No	Maybe	Low	High
1999, Tomori, DRC	Yes	Yes	Yes	No	Maybe	Yes	Yes	No	Maybe	Low	Moderate
1999, Bertherat, Gabon	Yes	Unclear	No	No	Maybe	Yes	Yes	No	No	Moderate	Low
1999, Jezek, DRC	Yes	Yes	Unclear	No	No	Maybe	Maybe	Maybe	Yes	Low	Low
1999, Rowe, DRC	Yes	Yes	Unclear	Maybe	Yes	Yes	Yes	No	Maybe	Low	Moderate
1999, Georges, Gabon	Yes	Yes	Yes	No	Maybe	Yes	Yes	No	Maybe	Low	Moderate
2000, Gonzalez, CAR	Unclear	Unclear	Yes	No	Maybe	Yes	Maybe	No	Maybe	Moderate	Low
2000, Nakounné, CAR	Yes	Yes	Yes	Maybe	Maybe	Yes	Yes	Maybe	Yes	Low	Moderate
2000, Leroy, Gabon	Yes	Yes	No	No	Yes	Yes	Yes	No	Yes	Low	Moderate
2003, Bausch, DRC	Yes	Unclear	Yes	Yes	Yes	Yes	Maybe	Yes	Maybe	Low	High
2005, Borchert, DRC	Yes	Unclear	Yes	Yes	Maybe	Yes	Yes	Yes	Maybe	Low	High
2005, Heffernan, Gabon	Unclear	Unclear	Yes	Yes	Maybe	Yes	Yes	Yes	Maybe	Low	Moderate
2006, Borchert, DRC	Unclear	Yes	Unclear	Yes	Maybe	Maybe	Maybe	Maybe	No	Low	Moderate
2006, Formenty, ROC	Yes	Unclear	No	No	No	Maybe	Yes	No	Maybe	Moderate	Low
2007, Lahm, Gabon	Unclear	Unclear	Yes	No	Maybe	Yes	Yes	No	Maybe	Low	Moderate
2007, Borchert, DRC	Yes	Yes	Unclear	Yes	Maybe	Unclear	Yes	No	Maybe	Low	High
2009, Wauquier, Gabon	Yes	Unclear	No	No	Maybe	Yes	Maybe	No	Yes	Moderate	Low
2010, Becquart, Gabon	Yes	Yes	Yes	Yes	Yes	Yes	Yes	Unclear	Yes	Very Low	High
2011, Nkoghe, Gabon[d]	Unclear	Unclear	Yes	Yes	Maybe	Yes	Yes	Yes	Maybe	Low	Moderate
2015, Moyen, ROC	Yes	Yes	Yes	Yes	Maybe	Unclear	Maybe	Maybe	Yes	Low	Moderate
2016, Mulangu, DRC	Unclear	Unclear	Yes	Yes	Maybe	Yes	Yes	Yes	Maybe	Low	Moderate
2017, Mbala, DRC	Unclear	Unclear	Yes	Maybe	Maybe	Yes	Yes	Maybe	Maybe	Low	Moderate

Year[a], Author, Location	Appropriateness / adequacy of:[b, c]									Risk of Bias Rating	GRADE Rating
	Sample frame	Participant sampling	Sample size	Description of setting & participants	Sample coverage for data analysis	Methods / measurements (validity)	Methods / measurements (standardized, reliable)	Statistical analysis	Response rate		
2018, Mulangu, DRC	Unclear	Unclear	Yes	Yes	Maybe	Yes	Yes	Maybe	Maybe	Low	Moderate
2019, Hoff, DRC	Unclear	Yes	Yes	Yes	Maybe	Yes	Yes	Maybe	Maybe	Low	Moderate
2020, Lucas, DRC	No	Yes	No	Yes	Maybe	Yes	Yes	No	Maybe	Low	Moderate
2020, Goldstein, DRC	Yes	Yes	Yes	Yes	Maybe	Yes	Yes	No	Maybe	Low	High
2021, Bratcher, DRC	Unclear	Unclear	Yes	Yes	Maybe	Yes	Yes	Yes	Maybe	Low	High
2022, Shaffer, DRC	Yes	Unclear	Yes	Yes	Yes	Unclear	Maybe	Yes	Yes	Very Low	High
2022, Nkuba-Ndaye, DRC	Unclear	Yes	Yes	Unclear	Maybe	Unclear	Maybe	Yes	Yes	Low	Moderate
2022, Doshi, DRC	Yes	Unclear	Yes	Yes	Maybe	Yes	Yes	Maybe	Yes	Low	Moderate
2023, Matuvanga, DRC	Yes	Unclear	Yes	Yes	Maybe	Yes	Yes	Yes	Yes	Very Low	High
**WEST AFRICA**											
1982, Knobloch, Liberia	Unclear	Unclear	Yes	No	Maybe	No	No	No	Maybe	Moderate	Low
1985, Van der Waals, Liberia	Yes	Yes	Yes	Unclear	Yes	No	No	No	Maybe	Low	Moderate
1988, Tomori, Nigeria	Unclear	Unclear	Yes	Unclear	Maybe	No	No	No	Maybe	Moderate	Moderate
2014, Schoepp, Multi	Yes	Yes	Maybe	No	Maybe	Unclear	Yes	No	Maybe	Low	Moderate
2015, Boisen, Sierra Leone	Unclear	Yes	Yes	No	Maybe	Yes	Yes	No	Yes	Low	Moderate
2016, O'Hearn, Sierra Leone	No	No	Yes	No	Maybe	Unclear	Maybe	Maybe	Maybe	Moderate	Moderate
2016, Richardson, Sierra Leone	Yes	Yes	Yes	Yes	No	Yes	Yes	No	Yes	Low	High
2016, Safronetz, Mali	Yes	Unclear	Yes	No	No	Unclear	Maybe	No	No	Moderate	Low
2017, Mafopa, Sierra Leone	Maybe	Unclear	Unclear	Maybe	No	Unclear	Yes	No	Maybe	Moderate	Low
2017, Glynn, Sierra Leone	Yes	Yes	Unclear	Yes	Maybe	Unclear	Maybe	Maybe	Maybe	Low	Moderate
2017, Houlihan, Sierra Leone	Unclear	Unclear	Yes	Yes	Maybe	Yes	Yes	Maybe	Maybe	Low	Moderate
2018, Kelly, Sierra Leone	Yes	Yes	Maybe	Yes	Maybe	Yes	Yes	Maybe	Yes	Low	Moderate
2018, Keita, Guinea	Unclear	Unclear	Yes	No	Maybe	Unclear	Maybe	Maybe	Maybe	Moderate	Low
2019, Diallo, Guinea	Yes	Yes	Yes	Yes	Maybe	Unclear	Maybe	Maybe	Maybe	Low	Moderate
2019, Halfmann, Sierra Leone	Unclear	No	Yes	No	Maybe	Yes	Maybe	No	Maybe	Moderate	Low
2019, Timothy, Guinea	Unclear	Unclear	Yes	Yes	Maybe	Unclear	Yes	Yes	Yes	Low	Moderate
2020, Surtees, Guinea	Unclear	Unclear	Unclear	No	Maybe	Unclear	Maybe	No	Maybe	Moderate	Moderate
2021, Bane, Mali	No	No	Yes	No	Maybe	Unclear	Yes	No	Maybe	Moderate	Low
2021, Kofman, Liberia	Yes	Yes	Unclear	Yes	Maybe	Yes	Yes	Maybe	Maybe	Low	Moderate
2021, Dedkov, Guinea	Yes	Unclear	Maybe	Yes	Maybe	Maybe	Maybe	Maybe	Maybe	Low	Moderate

Year[a], Author, Location	Appropriateness / adequacy of: [b, c]									Risk of Bias Rating	GRADE Rating
	Sample frame	Participant sampling	Sample size	Description of setting & participants	Sample coverage for data analysis	Methods / measurements (validity)	Methods / measurements (standardized, reliable)	Statistical analysis	Response rate		
2022, Manno, Sierra Leone	Yes	Yes	Yes	Yes	Maybe	Yes	Yes	Yes	Maybe	Very Low	High
2022a, Kelly, Sierra Leone	Yes	Yes	Yes	Yes	Maybe	Unclear	Maybe	No	Yes	Low	Moderate
2022b, Kelly, Liberia	Yes	Unclear	Yes	Yes	Maybe	Unclear	Yes	Maybe	Maybe	Low	Moderate
2023, Gayedyu-Dennis, Liberia[e]	Yes	Yes	Yes	Yes	Yes	Yes	Yes	No	Yes	Very Low	High
**SOUTHERN AFRICA**											
1978, Conrad, Zimbabwe	Unclear	Unclear	Unclear	No	Maybe	Maybe	Maybe	Unclear	Maybe	Moderate	Low
1982, Blackburn, Zimbabwe	Yes	Unclear	Yes	No	Maybe	No	No	No	Maybe	Moderate	Low
1987, Tessier, Botswana	Yes	Yes	Unclear	No	Yes	No	No	No	Yes	Low	Low
1989, Mathiot, Madagascar	Unclear	Unclear	Yes	No	Maybe	No	No	No	Maybe	Moderate	Low
**MULTI-REGION**											
1984, Slenczka, multi	Unclear	Unclear	Yes	No	No	No	No	No	Maybe	Moderate	Low
2019, Steffen, multi	Yes	Unclear	Yes	No	Maybe	Yes	Yes	Yes	Yes	Low	Moderate
2020, Steffen, multi	Yes	Unclear	Yes	No	Maybe	Maybe	Maybe	No	Maybe	Moderate	Low

Abbreviations: CAR, Central African Republic; DRC, Democratic Republic of Congo; GRADE, Grading of Recommendations, Assessment, Development, and Evaluations; ROC, Republic of Congo.

[a] Year of publication.

[b] Specific quality assessment questions were as follows:

Sample frame - Was the sample frame appropriate to address the target population?
Participant sampling - Were study participants sampled in an appropriate way?
Sample size - Was the sample size adequate?
Description of setting & participants - Were the study subjects and the setting described in detail?
Sample coverage for data analysis - Was the data analysis conducted with sufficient coverage of the identified sample?
Methods / measurements (validity) - Were valid methods used for the identification of the condition?
Methods / measurements (standardized, reliable) - Was the condition measured in a standard, reliable way for all participants?
Statistical analysis - Was there appropriate statistical analysis?
Response rate - Was the response rate adequate, and if not, was the low response managed appropriately

[c] In assessing appropriateness/adequacy for each domain, values of "Maybe" were treated as less adequate/appropriate than values of "Unclear" because in the case of "Unclear," a domain was addressed in some detail in the manuscript, but the adequacy or appropriateness of that domain

could not be determined from the description. On the other hand, in the case of “Maybe,” the domain was not mentioned or addressed adequately, although there also was no evidence that this domain had not been adequately addressed.
[d] This article describes the same study as the article listed above (Becquart, 2010).
[e] This article describes the same study as the article listed above (Kelly, 2022b).

Table S6: Assays used for detection of antibodies against orthoebolaviruses and orthomarburgviruses in antibody seroprevalence studies.

Year[a], Author, Location	Virus evaluated [b]	Type of assay[c]	Antibody isotype detected	Antigen / antibody target	Sample type	Definition for seropositivity	Comments
**EAST AFRICA**							
1982, Smith, Kenya	MARV	IFA	NS	Cells infected with MARV	Serum	Reactivity to monovalent infected (not uninfected) cells at ≥1:16 dilution	Little info provided on assay; Cites earlier study on IFA for LF
1982, Johnson, Kenya	EBOV MARV	IFA	NS	Cells infected with EBOV, MARV	Serum	Reactivity to monovalent infected (not uninfected) cells at ≥1:16 dilution	Little info provided on assay; Cites earlier study on IFA for LF
1983, Baron, Sudan	‘Ebola’[d] (assumed to be SUDV, since study in Sudan)	IFA	NS	NS	Serum	NS	Little info provided on assay; Cites to earlier study on IFA for LF
1983, Johnson, Kenya	EBOV SUDV MARV	IFA	NS	NS	Serum	Reactivity to monovalent infected (not uninfected) cells at ≥1:16 dilution	Little info provided on assay; Cites to earlier study on IFA for LF
1989, Rodhain, Uganda	EBOV SUDV MARV	IFA	NS	Cells infected with EBOV, SUDV, MARV	Serum	Reactivity to monovalent infected (not uninfected) cells at ≥1:10 dilution	Little info provided on assay; Cites to earlier study on IFA for LF
1993, Tignor, Ethiopia	EBOV MARV	IFA	NS	Cells infected with EBOV, MARV	Serum	Reactivity to monovalent infected (not uninfected) cells at ≥1:16 dilution	Little info provided on assay; Cites to earlier study on IFA for LF
2007, Onyango, Sudan	‘Ebola’[e] (Tested both EBOV & SUDV)	ELISA	IgG<br>IgM	Cells infected with EBOV, SUDV, RESTV	Serum	Either assay positive; Cutoff for each assay NS	None
2011, Adjemian, Uganda	MARV	ELISA	IgG	NS	NS	NS	None

Year[a], Author, Location	Virus evaluated[b]	Type of assay[c]	Antibody isotype detected	Antigen / antibody target	Sample type	Definition for seropositivity	Comments
2015, Clark, Uganda	BDBV<br>EBOV<br>SUDV<br>TAFV<br>MARV	ELISA	IgG	Cells infected with BDBV, EBOV, SUDV, MARV	Serum	Sample response > mean response +3SD of -ve controls for IgM and/or IgG	Plaque reduction neutralization tests were done on select sera to confirm the likely specificity of antibodies to BDBV
2015, Knust, Uganda	MARV	ELISA	IgG<br>IgM	NS	Serum	Sample response > mean + 3SD of -ve control samples	None
2019, Nyakarahuka, Uganda	MARV	ELISA	IgG<br>IgM	NS	NS	NS	Little info provided on assay; Cites earlier study on EBOV ELISA
2020, Nyakarahuka, Uganda	EBOV<br>SUDV<br>BDBV<br>MARV	ELISA	IgG	NS	Serum	Sample response of 1:400, 1:1600 or 1:6400 dilution was >0.2, & sum response >0.95	Assay validated by US CDC; Sens & spec reported as >90%; -ve & +ve control sera included in each assay run
2021, Rugarabamu, Tanzania	EBOV<br>MARV	ELISA (commercial)	IgG<br>IgM	NS	Serum	Ave OD of the -ve control wells + 0.15 (per manufacturer instructions)	Assay performance considered effective if intra-assay & inter-assay CV <15%
2022, Rugarabamu, Tanzania	EBOV<br>MARV	ELISA (commercial)	IgM	NS	Serum	Per manufacturer instructions	Within study determination of intra-assay CV, inter-assay CV, recovery, linearity, & parallelism performed; Intra-assay CV & inter-assay CV were <15% for reactive samples
**CENTRAL AFRICA**							
1978, Intl. Comm., DRC	EBOV	IFA	NS	Cells infected with EBOV	Serum	Reactivity to monovalent inrected (not uninfected) cells at ≥1:64 dilution	Little info provided on assay; Cites earlier study on IFA for LF
1979, Van der Groen, DRC	EBOV	IFA	NS	Cells infected with EBOV	Serum	Reactivity to monovalent inrected (not uninfected) cells at ≥1:16 dilution	Little info provided on assay; Cites earlier study on IFA for LF
1980, Heymann, DRC	EBOV	IFA	NS	Cells infected with EBOV	Serum	Reactivity to monovalent inrected (not uninfected) cells at ≥1:16 dilution	Little info provided on assay; Cites earlier study on IFA for LF
1980, Saluzzo, CAR	EBOV	IFA	NS	Cells infected with EBOV	Serum	NS	Little info provided on assay; Cites earlier study on IFA for LF
1982, Ivanoff, Gabon	EBOV<br>SUDV<br>MARV	IFA	NS	Cells infected with EBOV, SUDV, MARV	Serum	Reactivity to monovalent infected (not uninfected) cells at ≥ 1:16 dilution	Little info provided on assay; Cites earlier study on IFA for LF
1982, Talani, ROC	EBOV<br>MARV	IFA	NS	NS	Serum	NS	Little info provided on assay; Cites earlier study on IFA for LF
1983, Bouree, Cameroon	EBOV	IFA	NS	NS	Serum	Reactivity at ≥ 1:16 dilution	Little info provided on assay; Cites earlier study on IFA for LF

Year[a], Author, Location	Virus evaluated [b]	Type of assay[c]	Antibody isotype detected	Antigen / antibody target	Sample type	Definition for seropositivity	Comments
1987a, Meunier, CAR	EBOV	IFA	NS	Cells infected with EBOV	Serum	Reactivity to monovalent infected (not uninfected) cells at ≥ 1:16 dilution	Little info provided on assay; Cites earlier study on IFA for LF
1987b, Meunier, Gabon	EBOV SUDV MARV	IFA	NS	Cells infected with EBOV, SUDV, MARV	Serum	Reactivity to monovalent infected (not uninfected) cells at ≥ 1:16 dilution	Little info provided on assay; Cites earlier study on IFA for LF
1988, Paix, Cameroon	EBOV MARV	IFA	NS	NS	Serum	Reactivity to monovalent infected (not uninfected) cells at ≥ 1:16 dilution	Little info provided on assay; Cites earlier study on IFA for LF
1989, Gonzalez, multi	'Ebola'[e] (Tested both EBOV & SUDV) MARV	IFA	NS	Cells infected with EBOV, SUDV, MARV	Serum	NS	Cites earlier study on IFA for LF; Samples 1st tested on polyvalent slides (6 viruses); Broadly reactive samples tested on monovalent slides
1993a, Johnson, CAR	EBOV SUDV MARV	IFA	NS	Cells infected with EBOV, SUDV, MARV	Serum	Reactivity to monovalent infected (not uninfected) cells at ≥ 1:16 dilution	Samples first tested on polyvalent slides (6 viruses); Broadly reactive samples tested on monovalent slides
1993b, Johnson, CAR	EBOV SUDV MARV	IFA	NS	Cells infected with EBOV, SUDV, MARV	Serum	Reactivity to monovalent infected (not uninfected) cells at ≥ 1:128 dilution	Samples first tested on polyvalent slides (6 viruses); Broadly reactive samples tested on monovalent slides
1999, Bertherat, Gabon	EBOV	ELISA	IgG<br>IgM	Cells infected with EBOV	Serum	Sample response > mean + 2SD of 3 -ve control samples<br>NS	Little info provided on assay; Cites earlier study on IFA for LF
1999, Busico, DRC	EBOV	ELISA	IgG	EBOV antigen – details not provided	Serum	Antibody titer ≥ 1:400 & summed OD ≥ 1.25	EBOV response corrected for negative control antigen response for each serum sample; Cites earlier study for more assay details
1999, Tomori, DRC	EBOV	ELISA	IgG<br>IgM	Cells infected with EBOV	Serum	Sum of adjusted optical densities for 4 dilutions tested >1.25 for IgG or >0.50 for IgM	4 of the 12 positive samples later tested negative for IgG
1999, Jezek, DRC	SUDV EBOV	IFA	NS	Cells infected with EBOV, SUDV	Serum	Sample response above cutoff (titer ≥ 1:64)	Virus isolation using Vero cell tissue culture or in suckling mice planned but not done because of bacterial contamination of specimens; Cites earlier study for more assay details
1999, Rowe, DRC	EBOV	ELISA	IgG<br>IgM	Cells infected with EBOV	Blood	Sample response > mean response +3SD of -ve controls	RT-PCR was also done to detect presence of active virus in contacts. However, for patients that had ELISA performed, if they were ever positive, they were indicated as EBOV seropositive
	EBOV	ELISA	IgG		Serum		None

Year[a], Author, Location	Virus evaluated [b]	Type of assay[c]	Antibody isotype detected	Antigen / antibody target	Sample type	Definition for seropositivity	Comments
1999, Georges, Gabon		IFA	IgM	Cells infected with EBOV		Sample response > mean response +3SD of -ve controls	
2000, Nakounné, CAR	EBOV<br>MARV	ELISA<br>IFA	IgG	Cells infected with EBOV & MARV	Serum	Reactivity to monovalent infected (not uninfected) cells at ≥ 1:400 dilution	None
2000, Gonzalez, CAR	EBOV<br>MARV	ELISA	IgG	Cells infected with EBOV, MARV	Serum	Titer ≥ 1:400 & summed OD of all 4 dilutions >1.000. Cutoff = mean + 2SD from known -ve control	Cites earlier study for more assay details
2000, Leroy, Gabon	EBOV	ELISA	IgG<br>IgM	Cells infected with EBOV	Serum	Mean adjusted optical density for 10 normal serum samples (+350)	None
2003, Bausch, DRC	MARV	ELISA<br>*IFA*	IgG<br>IgM	Cells infected with MARV	Serum	Sample response > 3SD from mean control adjusted response<br>Sample response above cutoff (1:50)	In ELISA, cutoff calculated using panel of 'normal' sera. African patients with & without lab-confirmed MD used as +ve & -ve controls. All ELISA+ samples tested by IFA. Samples considered reactive only if +ve in both assays; Cites earlier study for more assay details
2005, Borchert, DRC	MARV	ELISA<br>IFA	IgG	Cells infected with MARV	NS	Both assays positive; Cutoff for each assay NS	Little information provided on assay and methods provided but cites earlier study for more details
2005, Heffernan, Gabon	EBOV	ELISA	IgG	Cells infected with EBOV, SUDV, RESTV	Serum	NS	IgG+ samples & random selection of IgG- samples retested at US CDC; Cites earlier study for more assay details
2006, Borchert, DRC	MARV	ELISA<br>IFA	IgG	NS	Serum	Both assays positive; Cutoff for each assay NS	None
2006, Formenty, ROC	EBOV	ELISA	IgG	Cells infected with EBOV	Serum	NS	Assay also evaluated IgG detection in oral fluid but results not presented here; Cites earlier study for more assay details
2007, Lahm, Gabon	EBOV	ELISA	IgG<br>IgM	Cells infected with EBOV	Serum	Sample response > mean response +3SD of -ve controls	None
2007, Borchert, DRC	MARV	ELISA<br>IFA	IgG	Cells infected with MARV	Serum	Both assays positive; Cutoff for each assay NS	Little information provided on assay and methods provided but cites earlier study for more details (albeit EBOV assay for ELISA)
2009, Wauquier, Gabon	EBOV	ELISA	IgG	Cells infected with EBOV, SUDV, RESTV	Plasma	Sample response > mean response +3SD of -ve controls	Sera from 103 Caucasians living in Europe used as -ve controls for cut-off calculation; Cites earlier study for more assay details

Year[a], Author, Location	Virus evaluated [b]	Type of assay[c]	Antibody isotype detected	Antigen / antibody target	Sample type	Definition for seropositivity	Comments
2010, Becquart, Gabon	EBOV	ELISA	IgG	Cells infected with EBOV, SUDV, RESTV	Plasma	Sample response > cutoff (error risk <1/1000 for 1st "+ve" value)	Sera from 104 French individuals who had never visited Africa used as -ve controls for cut-off calculation; Cites earlier study for more assay details
2011, Nkoghe, Gabon[f]	EBOV	ELISA	IgG	Cells infected with EBOV, SUDV, RESTV	Plasma	Sample response > cutoff (error risk <1/1000 for 1st "+ve" value)	Sera from 104 French individuals who had never visited Africa used as -ve controls for cut-off calculation; Cites earlier study for more assay details
2015, Moyen, ROC	EBOV<br>MARV	IFA	IgG	Cells infected with EBOV, MARV	Serum	Sample response > cutoff (end point titers ≥ 20)	Cites earlier study for more assay details
2016, Mulangu, DRC	EBOV	ELISA	IgG	NS	Serum	Sample response > 2x [mean + 3SD] of -ve controls	Cutoff calculated used South African controls assumed to be seronegative; Cites earlier study for more assay details
2017, Mbala, DRC	EBOV	ELISA	IgG<br>IgM	GP-EBOV	Serum	Sample response to GP-EBOV minus sample response to -ve control antigen ≥ 0.95	Cites earlier study for more assay details
2018, Mulangu, DRC	EBOV	ELISA	IgG	EBOV-NP	Serum	Sample response ≥ 5SD from -ve control mean	Cutoff was based on ave response of 88 donors from Kinshasa; Reports 89% sens, 99% spec, 94% PPV, 98% NPV for assay
2019, Hoff, DRC	EBOV	ELISA (commercial)	IgG	GP-EBOV<br>NP-EBOV	Serum	Sample response > cutoff (2.5 U/ml)	Sample considered seroreactive if +ve in ≥1 assay; For luciferous IP system, background determined from mean of 8 prev. identified VP40 -ve samples.
		Luciferase IPA	-	VP40-EBOV		Sample response > 3 SD above background	
		Neutralization assay	-	EBOV GP		Sample neutralized ≥ 50% of virus at 1:50 dilution compared to control	
2020, Lucas, DRC	EBOV<br>*SUDV*<br>*BDBV*<br>*TAFV*<br>*MARV*	ELISA	IgG	GP-EBOV<br>*GP-SUDV*<br>*GP-BDBV*<br>*GP-TAFV*<br>*GP-MARV*	Serum	Sample response > 3x background or -ve controls	Reactive samples on EBOV assay screened against other filoviruses due to potential for cross-reactivity
2020, Goldstein, DRC	EBOV<br>*SUDV*<br>*BDBV*<br>*RESTV*<br>*MARV*	ELISA	IgG	GP-EBOV<br>*GP-SUDV*<br>*GP-BDBV*<br>*GP-RESTV*<br>*GP-MARV*	Serum	Sample response > 3x background or -ve controls	Sera 1st screened for reactivity to GP-EBOV; +ve samples tested for reactivity to all filovirus GPs; EBOV sero+ & sero- human serum samples from Uganda used as controls
2021, Bratcher, DRC	EBOV	ELISA (commercial)	IgG	GP-EBOV	Serum	Sample response > cutoff (4.7 unit/ml)	Cutoff higher than that recommended by manufacturer, as suggested by earlier study; Reports 97% sens & 98% spec for assay
2022, Nkuba-Ndaye, DRC	EBOV	Multiplex (Luminex)	IgG	2x GP-EBOV<br>NP-EBOV<br>VP40-EBOV	Serum	Sample response for ≥ 2 EBOV antigens; Cutoff NS	Cites earlier study for more assay details

Year[a], Author, Location	Virus evaluated[b]	Type of assay[c]	Antibody isotype detected	Antigen / antibody target	Sample type	Definition for seropositivity	Comments
2022, Shaffer, DRC	EBOV<br>SUDV<br>BDBV	ELISA	IgG	GP-EBOV<br>GP-SUDV<br>GP-BDBV	Serum	Sample response > EC50 of positive control	Non-immune pooled sera from African individuals served as -ve control; Pan-ebolavirus monoclonal antibody isolated from ED survivor served as +ve control
2022, Doshi, DRC	EBOV	ELISA (commercial)	IgG	GP-EBOV	Serum	Sample response > cutoff (≥ 2.5 units/ml)	Cutoff higher than that recommended by manufacturer, as suggested by earlier studies.
2023, Matuvanga, DRC	EBOV	ELISA (FANG)	IgG	GP-EBOV	Serum	Sample response > cutoff (526 EU/ml)	Study conducted per GLP; Absence of established cutoffs for assays, so calculated for study using change point analysis; Samples tested with both assays (poor concordance); Reported 97% sens & 95% spec for ELISA; Cites earlier study for more assay details
		Multiplex (Luminex)		2x GP-EBOV<br>NP-EBOV<br>VP40-EBOV		Sample response > cutoff (669 MFI/100 beads) for ≥ 2 EBOV antigens	
**WEST AFRICA**							
1982, Knobloch, Liberia	EBOV<br>MARV	IFA	IgG	Cells infected with EBOV, MARV	Serum	NS	Little info provided on assay; Cites earlier study on IFA for LF
1985, Van der Waals, Liberia	EBOV<br>SUDV<br>MARV	IFA	NS	Cells infected with EBOV, SUDV, MARV	Serum	Reactivity to monovalent infected (not uninfected) cells at ≥ 1:8 dilution, then screened individually on monovalent antigens and diluted two-fold	Little info provided on assay; Cites earlier study on IFA for LF
1988, Tomori, Nigeria	SUDV<br>*EBOV*	IFA	NS	Cells infected with SUDV, EBOV	Serum	Reactivity to monovalent infected (not uninfected) cells at ≥ 1:10 dilution	Little info provided on assay; Cites earlier study on IFA for LF
2014, Schoepp, multi Sierra Leone	EBOV<br>*SUDV*<br>*TAFV*<br>MARV	ELISA	IgG<br>IgM	Cells infected with EBOV, MARV	Serum	Sample response > mean response +3SD of -ve controls for IgM and/or IgG	Due to potential for cross-reactivity, PRNT conducted on EBOV IgM +ve samples (where enough sample available) using EBOV, SUDV & TAFV; Cites earlier study for more assay details, but on assays for RVFV & VEE
		Neutralization assay	-	EBOV, SUDV, TAFV		Sample response > cutoff (titer ≥ 10)	
2015, Boisen, Sierra Leone	EBOV	ELISA	IgG<br>IgM	GP-EBOV<br>VP40-EBOV	Serum	Optical density at 450 nm of 0.360	Higher background reactivity in older samples, indicating that temperature fluctuations during storage could have caused sample degradation and assay background
2016, O'Hearn, Sierra Leone	EBOV<br>MARV	Multiplex (Luminex)	IgG	GP-EBOV<br>VP40-EBOV<br>VP40-MARV	Serum	Sample response > cutoff (z-score ≥ 3SE > 0) for ≥1 viral antigen	Test results with readings from <10 beads per well considered unreliable & excluded from analysis; Cites earlier study for more assay details
2016, Safronetz, Mali	EBOV	ELISA (commercial)	IgG<br>IgM	NS	Serum	Sample response > 3 SD above background or average of known -ve controls	None

Year[a], Author, Location	Virus evaluated[b]	Type of assay[c]	Antibody isotype detected	Antigen / antibody target	Sample type	Definition for seropositivity	Comments
2016, Richardson, Sierra Leone	EBOV	ELISA (commercial)	IgG	GP-EBOV	Plasma	Sample response > cutoff (4.7 U/ml)	Assay validated in study using plasma from PCR-confirmed ED survivors & controls with minimal likelihood of EBOV exposure; Cutoff & diagnostic accuracy established by ROC analysis (sens 97%, spec 98%)
2017, Houlihan, Sierra Leone	EBOV	IgG capture assay	IgG	GP-EBOV	Oral fluid	Sample response > cutoff (mean response for -ve controls + 0.1)	Use of oral swabs validated against plasma with good concordance; 96% sens & 100% spec reported for assay
2017, Glynn, Sierra Leone	EBOV	IgG capture assay	IgG	GP-EBOV	Oral fluid	Sample response > mean response for -ve controls + 0.1 in 2 tests	Study validated assay prior to seroprev assessment (sens 96%, spec 100%); Use of oral swabs validated against plasma with good concordance
2017, Mafopa, Sierra Leone	EBOV	ELISA (commercial)	IgG	NP-EBOV	Plasma	Calculated in study but NS (see comments)	+ve control & calibrators used in each test run; Threshold index for +ve response calculated following manufacturer instructions
2018, Keita, Guinea	EBOV	Multiplex (Luminex)	IgG	GP-EBOV<br>NP-EBOV<br>VP40-EBOV	DBS	Sample response simultaneously & repeatedly to NP & GP; cutoff NS	Cutoff determined with ROC curve analysis with EBOV -ve & +ve plasma; Algorithm to determine +ve response defined using survivors of Guinean Ebola outbreak; Cites earlier study for more assay details
2018, Kelly, Sierra Leone	EBOV	ELISA (commercial)	IgG	GP-EBOV<br>NP-EBOV	NS	Sample response > cutoff (4.7 U/ml)	Cites earlier study for more assay details
2019, Timothy, Guinea	EBOV	ELISA (commercial)	IgG	GP-EBOV	Oral fluid	NS	Oral swabs from 2 PCR +ve ED survivors used as positive controls; Cites earlier study for more assay details
2019, Diallo, Guinea	EBOV	Multiplex (Luminex)	IgG	GP-EBOV<br>NP-EBOV<br>VP40-EBOV	DBS	Sample response > cutoff (GP: 501MFI, NP: 950 MFI, 40kDa VP: 580 MFI) for ≥2 antigens	Cutoff thresholds defined previously; Reported sens & spec >90% for assay; Earlier validation based on samples from ED survivors
2019, Halfmann, Sierra Leone	EBOV	ELISA (commercial)	IgG	GP-EBOV<br>NP-EBOV<br>VP40-EBOV	Plasma	Sample response > cutoff (see comments) for ≥1 antigen.	Using calibrators provided in ELISA kits, threshold index established for each ELISA run for +ve vs. -ve response, but details not provided
2020, Surtees, Guinea	EBOV<br>MARV	Multiplex (microsphere)	IgG	NP-EBOV<br>NP-MARV	Serum	Sample response > 99th percentile for responses in German blood bank population	Article describes assay development, validation, and application in Guinean samples; Good agreement with in-house ELISA
2021, Kofman, Liberia	EBOV	ELISA	IgG<br>IgM	GP-EBOV<br>NP-EBOV	Serum	Adjusted OD of > 0.2 required for dilution to be considered positive and ≥ 1:400 titer and sum of adjusted OD ≥ 0.95	None
2021, Bane, Mali	EBOV	ELISA (commercial)	IgG	GP-EBOV<br>NP-EBOV	Serum	Sample response at ≥1:400 dilution; Cutoff NS	Sample response at ≥1:100 dilution considered equivocal

Year[a], Author, Location	Virus evaluated[b]	Type of assay[c]	Antibody isotype detected	Antigen / antibody target	Sample type	Definition for seropositivity	Comments
2021, Dedkov, Guinea	EBOV MARV	IgM microarray	IgM	NP-ZEBOV NP-MARV	Serum	Sample response > cutoff (5 µg/ml)	Earlier validation work cited for other viruses evaluated through microarray, but not EBOV or MARV; Reported 100% & 98% spec for EBOV assay, & 98% spec for MARV assay
2022, Manno, Sierra Leone	EBOV	ELISA (FANG)	IgG	GP-EBOV	Serum	Sample response > cutoff (607 EU/ml)	Validation of FANG ELISA endorsed by US FDA in Feb 2017; Absence of established cutoffs, so used cutoff established in earlier study; Cites earlier study for more assay details
2022a, Kelly, Sierra Leone	EBOV	ELISA (FANG)	IgG	GP-EBOV	Plasma	Sample response > cutoff (548 EU/ml)	94% sens & 97% spec reported for assay
2022b, Kelly, Liberia	EBOV	ELISA (FANG)	IgG	GP-EBOV	Serum	Sample response > cutoff (548 EU/ml)	94% sens & 97% spec reported for assay
2023, Gayedyu-Dennis, Liberia[g]	EBOV	ELISA (FANG)	IgG	GP-EBOV	Serum	Sample response > cutoff (548 EU/ml)	94% sens & 97% spec reported for assay
**SOUTHERN AFRICA**							
1978, Conrad, Zimbabwe	MARV	IFA	NS	Cells infected with MARV	Serum	NS	Little info provided on assay; Cites earlier study on IFA for LF
1982, Blackburn, Zimbabwe	EBOV MARV	IFA	NS	NS	Serum	Reactivity at ≥ 1:16 dilution	Little info provided on assay; Cites earlier study on IFA for LF
1987, Tessier, Botswana	EBOV SUDV MARV	IFA	NS	Cells infected with EBOV, SUDV, MARV	Serum	Reactivity to monovalent infected (not uninfected) cells at ≥1:16 dilution	Little info provided on assay; Cites earlier study on IFA for LF
1989, Mathiot, Madagascar	EBOV SUDV MARV	IFA	NS	Cells infected with EBOV, SUDV, MARV	Serum	Reactivity to monovalent infected (not uninfected) cells at ≥1:16 dilution	Little info provided on assay; Cites earlier study on IFA for LF
**MULTI-REGION**							
1984, Slenczka, multi	EBOV SUDV MARV	Enzyme immunoassay	IgG	Cells infected with EBOV, SUDV, MARV	Serum	Positivity against EBOV, SUDV, or MARV at a dilution of 1:100	The enzyme immunoassay was a piloted assay that was validated using IFA results. Little assay provided on IFA; Cites earlier study on IFA for LF
		IFA	NS			Reactivity to monovalent infected (not uninfected) cells at ≥1:64 dilution	
2019, Steffen, multi	EBOV	ELISA (commercial)	IgG	EBOV-NP	Serum	Sample response > cutoff (4.62 U/ml)	All assays incl. sera from EBOV survivors as +ve controls; For neutralization assay, Pseudotypes with GP-MACV incl. as -ve control; For Luciferous IPA, background response used 10 -ve samples from Kinshasa. For ELISA, used 47 samples from Kinshasa to determine background reactivity; Serum samples reactive in ≥2 assays considered seroreactive
		Neutralization assay	-	EBOV-GP		Sample reduced infectivity of EBOV pseudotypes by >50%	
		Luciferase IPA assay	-	VP40-EBOV		NS	

Year[a], Author, Location	Virus evaluated [b]	Type of assay[c]	Antibody isotype detected	Antigen / antibody target	Sample type	Definition for seropositivity	Comments
2020 Steffen, multi	MARV	Neutralization assay	-	GP-MARV	Serum	Sample reduced infectivity of MARV pseudotypes by >50%	Pseudotypes with GP-MACV incl. as spec. control; ELISA conducted for samples reactive in PNA; For ELISA, used samples from similar locality to determine background reactivity; Serum samples reactive in both assays considered seroreactive
		ELISA				Sample response > ave + 3SD of background for each run	

Abbreviations: ave, average; BDBV, Bundibugyo virus; CAR, Central African Republic; CCHF, Crimean–Congo hemorrhagic fever; CV, coefficient of variation; DBS, dried blood spot; DRC, Democratic Republic of Congo; EBOV, Ebolavirus; EC50, half maximal effective concentration; ELISA, enzyme-linked immunosorbent assay; EU, endotoxin units; ED, Ebola disease; FANG, Filovirus Animal Non-Clinical Group; GP, glycoprotein; IFA, immunofluorescence assay; IgG, immunoglobulin G; IgM, immunoglobulin; IPA, immunoprecipitation assay; kDa, kilodalton; LF, Lassa Fever; MACV, Machupo virus; M; MARV, Marburgvirus; MFI, median fluorescent intensity; MD, Marburg disease; ml, milliliter; nm, nanometer; NS, not stated; NP, nucleoprotein; OD, optical density; PCR, polymerase chain reaction; PNA, pseudovirus neutralization assay; PRNT, plaque reduction neutralization test; RESTV, Reston virus; RVFV, Rift valley fever virus; ROC, Republic of Congo; ROC curve, receiver operating characteristic curve; SD, standard deviation; SUDV, Sudan virus; TAFV, Taï Forest virus; U, unit; US CDC, United States Centers for Disease Control and Prevention; VEE, Venezuelan equine encephalitis; VP40, viral protein 40; +ve, positive; -ve, negative; µg, microgram.

[a] Year of publication.

[b] Viruses written in italics were only evaluated among participants who tested EBOV antibody seropositive.

[c] Assays written in italics were only used for samples where the primary assay yielded a positive result.

[d] This article did not provide details of the antigen used in the assay and just described measurement of antibodies to 'Ebola', but the study was conducted in an area affected by a SUDV outbreak.

[e] These studies used an assay that measures antibody responses to EBOV and SUDV simultaneously, and therefore did not present results for each specific virus.

[f] This article describes the same study as the article listed above (Becquart, 2010).

[g] This article describes the same study as the article listed above (Kelly, 2022b).
